# Global HIV Incidence Analysis and Implications for Affordability Using Long-Acting Cabotegravir Versus Continuous and Event-Driven Oral Preexposure Prophylaxis

**DOI:** 10.1093/cid/ciad537

**Published:** 2023-09-04

**Authors:** Ishani Sharma, Andrew Hill

**Affiliations:** School of Medicine, Imperial College London, London, UK; Department of Pharmacology and Therapeutics, University of Liverpool, Liverpool, UK

**Keywords:** HIV prevention, preexposure prophylaxis, cabotegravir, tenofovir/emtricitabine, event-driven PrEP

## Abstract

**Background:**

The HIV Prevention Trials Network (HPTN) 083/084 trials showed up to 88% increased efficacy of long-acting cabotegravir (CAB-LA) versus continuous oral tenofovir disoproxil fumarate/emtricitabine (TDF/FTC). However, CAB-LA's high price limits the number of people who can be treated within fixed prevention budgets. Global human immunodeficiency virus (HIV) prevention budgets are highly limited, with TDF/FTC widely available as a low-cost generic. In randomized clinical trials, event-driven TDF/FTC has shown similar preventive efficacy to continuous TDF/FTC.

**Methods:**

A systematic review of global HIV incidence studies was conducted. Weighted incidence was calculated in each at-risk population. HIV infection rates were evaluated for 5 prevention strategies, with additional HIV testing, education, and service access costs assumed for each ($18 per person per year). Assumed efficacies were 90% (continuous CAB-LA), 60% (continuous TDF/FTC), and 60% (event-driven TDF/FTC). Using weighted incidence and an assumed 100 000 target population, annual HIV infection rates by population were calculated for each prevention strategy.

**Results:**

Ninety-eight studies in 5 230 189 individuals were included. Incidence per 100 person-years ranged from 0.03 (blood donors) to 3.82 (people who inject drugs). Using the number needed to treat to benefit for each strategy, a mean incidence of 2.6 per 100 person-years in at-risk populations, and a 100 000 target population, current-price continuous CAB-LA cost $949 487 per HIV infection successfully prevented, followed by target-price CAB-LA ($11 453), continuous TDF/FTC ($4231), and event-driven TDF/FTC ($1923).

**Conclusions:**

High prices of CAB-LA limit numbers treatable within fixed budgets. Low-cost event-driven TDF/FTC consistently prevents the most HIV infections within fixed budgets.

## Teaser

This systematic review and analysis investigated HIV incidence in at-risk populations globally. Analysis of 5 prevention strategies found that, across all populations, event-driven tenofovir/emtricitabine was cheapest per infection prevented, thus could avert more infections within budget, despite a lower number needed to treat to benefit.

The human immunodeficiency virus (HIV) epidemic continues to grow, with 1.5 million new infections in 2021 [1]. In 2019, 62% of these were among key populations, including men who have sex with men (MSM), transgender women (TGW), people who inject drugs (PWID), and commercial sex workers (CSWs), as well as their partners [2, 3].

Adolescent girls and young women (AGYW) and pregnant women (PW) are also key foci. For AGYW, this is due to multiple transmission risks, particularly via gender-based violence (GBV), meaning they could benefit from preexposure prophylaxis (PrEP) and postexposure prophylaxis [4]. For PW, this is due to vertical transmission risk [5] and ease of incorporating HIV diagnostics and prevention into antenatal healthcare.

Multiple HIV prevention strategies are available: Combination antiretroviral PrEP is well-established, including continuous tenofovir disoproxil fumarate/emtricitabine (TDF/FTC), a daily oral medication. Newer event-driven therapies also use oral TDF/FTC, taken “on-demand” when patients anticipate HIV exposure [6]. Recently, long-acting cabotegravir (CAB-LA), a continuous injectable PrEP regimen, was also licensed.

Table 1 summarizes key PrEP trials. Different PrEP strategies showed mixed results: Continuous oral TDF/FTC has been well-established as efficacious in populations where it is more socially acceptable and used correctly [7]. However, trials reported variable adherence to continuous TDF/FTC across different settings and populations, for reasons including daily administration, stigma, and “PrEP shaming” following disclosure, based on PrEP-user stereotyping as “promiscuous” or “immoral” [8–10].

**Table 1.** Key Preexposure Prophylaxis Trials Conducted From 2010 Onward

Type of PrEP (Area of Study)	Current Cost of PrEP, US$ pppy	Trial Name (Year)	Risk Population	Key Findings
Continuous TDF/FTC (international)	$48 [11]	iPrEx (2010) [12]	MSM and TGW	Daily oral TDF/FTC treatment resulted in a 44% risk reduction in HIV incidence
		TDF2 (2012) [13]	Heterosexual men and women	Daily oral TDF/FTC treatment resulted in a 62% risk reduction in HIV incidence
		PartnersPrEP (2012) [14]	Serodiscordant heterosexual couples	Daily oral TDF/FTC treatment resulted in a 75% risk reduction in HIV incidence
		PROUD (2016) [15]	MSM	86% risk reduction of HIV incidence in groups using daily oral TDF/FTC
Event-driven TDF/FTC (France)	$12 [11, 16]	IPERGAY (2015) [17]	MSM	86% risk reduction in HIV incidence groups using event-driven TDF/FTC
Continuous and event-driven TDF/FTC (France)	$48 (continuous); $12 (event-driven) [11, 16]	PREVENIR (2022) [18]	MSM and TGW	No difference in HIV incidence between groups using continuous or event-driven PrEP
Continuous CAB-LA (international)	$22 200 [19] ($250 target price [20])	HPTN 083 (2021) [21]	MSM and TGW	66% reduction in HIV infections using CAB-LA vs TDF/FTC
		HPTN 084 (2022) [22]	18- to 45-year-old women	88% reduction in HIV infection using CAB-LA vs TDF/FTC

Abbreviations: CAB-LA, long-acting cabotegravir; HIV, human immunodeficiency virus; HPTN, HIV Prevention Trials Network; IPERGAY, intervention préventive de l’Exposition aux risques avec et pour les gays ; iPrEx, preexposure prophylaxis initiative ; MSM, men who have sex with men; PrEP, preexposure prophylaxis; PREVENIR, l’Agence française de recherche sur le sida prévenir ; PROUD, pre-exposure option for reducing HIV in the UK ; pppy, per person per year; TDF2, botswana TDF/ FTC oral HIV prophylaxis trial ; TDF/FTC, tenofovir disoproxil fumarate/emtricitabine; TGW, transgender women; US$, United States dollars.

The pre-exposure option for reducing HIV in the UK (PROUD) and intervention préventive de l’Exposition aux risques avec et pour les gays (IPERGAY) studies highlighted the comparable efficacies of continuous and event-driven PrEP, each reducing HIV incidence by 86% [15, 17]. The l’Agence française de recherche sur le sida prévenir (PREVENIR) study corroborated this, showing no significant difference in HIV incidence between groups using continuous or event-driven PrEP [18].

Studies suggest that CAB-LA is more efficacious than TDF/FTC and expands nonoral PrEP options [21, 22]. CAB-LA also addresses some adherence barriers. For example, the 2-monthly injections are administered discretely in clinic, resolving issues of remembering a daily medication and providing some privacy to address concerns surrounding disclosure [23].

However, CAB-LA costs $22 200 per person per year (pppy) in the United States and $9275 pppy in the United Kingdom, despite a $250 pppy target price and an estimated generic price of $16–$34 pppy [11, 19, 20, 24]. The voluntary licensing agreement between the CAB-LA manufacturer, ViiV, and the Medicines Patent Pool leaves many countries without affordable CAB-LA, despite high HIV incidence [19]. It is unclear what the final generic CAB-LA prices will be or when they will become available under this voluntary license.

Furthermore, in-clinic administration medicalizes CAB-LA, requiring trained healthcare staff for regular injections, as CAB-LA is not available as an event-driven regimen, incurring further costs. Rather than prioritizing HIV prevention access in the community, reliance of CAB-LA–based prevention on central healthcare access and engagement presents a potential adherence barrier.

The number needed to treat for benefit (NNTB) for CAB-LA must also be considered. NNTB varies; however, even at a lower estimated NNTB of 30, preventing 1.5 million new infections requires 45 million people to receive CAB-LA. This would cost almost $1 billion (medication only), and in 2021, only 1.6 million people accessed PrEP [1]. Furthermore, evidence suggests that CAB-LA increases resistance risk to integrase stand transfer inhibitors, including dolutegravir, which are first-line HIV treatments [25].

Table 2 shows recorded PrEP spending by country, highlighting global variability. Given fixed annual PrEP finances, many countries may find current-price CAB-LA unaffordable, compared to generic continuous TDF/FTC ($48 pppy), which is used widely including in Europe and Central Asia [11, 26].

**Table 2.** Top 10 Low- and Middle-Income Countries by Preexposure Prophylaxis Spending (US$), and Annual Infections for the Most Recent Year Recorded

Country (Year Recorded)	PrEP Spending, US$	Annual Infections, No.
Zimbabwe (2020)	98 152 317	25 000
South Africa (2020)	14 978 901	220 000
Brazil (2023)	6 000 000	920 000
Kenya (2021)	4 324 962	35 000
Thailand (2021)	2 605 157	520 000
Malawi (2021)	1 598 000	20 000
Dominican Republic (2021)	1 271 961	4200
Ukraine (2021)	230 825	240 000
Bangladesh (2021)	143 253	1100
Kazakhstan (2021)	119 722	3500

Sources: Joint United Nations Programme on HIV/AIDS and International AIDS Society [30, 31.

Abbreviations: PrEP, preexposure prophylaxis; US$, United States dollars.

Comparison of these PrEP strategies in at-risk populations, particularly considering efficacy and financial budgets, is currently limited. Therefore, this research investigates HIV risk in populations that could benefit from PrEP and explores affordability of these PrEP regimens to discern the best strategy to tackle worsening HIV epidemics.

## METHODS

### Search Strategy

This systematic review was conducted following the Cochrane framework for systematic reviews and reported according to the Preferred Reporting Items for Systematic Reviews and Meta-Analyses (PRISMA) guidelines [27].

To determine HIV incidence in key at-risk populations of focus globally, a systematic review of literature from 2018 to the present was conducted on 3 Ovid databases (Medline, Embase, and Global Health).

Key populations were MSM, TGW, CSWs, PWID, PW, and AGYW. Blood donors and the general population were included as comparators.

Cochrane Review was searched to identify key terms used in other literature searches for each of the themes “HIV,” “incidence,” and “key populations.” No language limitations were used. The full search strategy is shown in Supplementary Appendix 1.

### Screening

From the 3 databases, 13 733 titles and abstracts were screened, and 379 were included for full-text screening using COVIDence Software (after duplicate removal). Forty-one articles from the supplementary search and reference screening were also included for full-text screening.

Population, intervention, comparator, outcomes, study (PICOS) inclusion and exclusion criteria are summarized in Supplementary Appendix 2 and were used to determine the eligibility of articles for the research question. Included studies reported incidence and sample size of risk populations, or in general populations/blood donors for comparison. Studies using recency testing were included due to the well-evidenced accuracy of recency assays in detecting new infections, alongside studies using other forms of HIV testing including enzyme-linked immunosorbent assays [28].

Incidence modeling studies were excluded due to variability in model parameters resulting in difficulty in comparing different model results. Intervention-based randomized controlled trials were excluded due to highly controlled participant recruitment, meaning trial groups may not be representative of the entire key population.

Ninety-eight studies were included for final data extraction and analysis, included in Supplementary Appendix 3. Supplementary Appendix 4 shows a PRISMA flowchart detailing review methodology and reasons for exclusion.

### Quality Assessment and Data Extraction

The quality of included studies was assessed using the Critical Appraisal Skills Program (CASP) Framework to ensure high quality. Studies were scored with a quality of high (CASP score >8), moderate (CASP score 5–8 inclusive), or low (CASP score <5) [29].

Data from 33 countries and all key populations were included for data analysis. All CSWs included were female (referred to as “FSWs” for the remainder of this review). Incidence data were extracted and converted to an incidence rate per 100 person-years (PY). The weighted average incidence was calculated for each population, blood donors, and the general population using study population size.

### Implications for Affordability

Using calculated weighted mean HIV incidences, affordability was analyzed for different PrEP regimens on HIV in key populations. Annual numbers of HIV infections successfully prevented and infections remaining in a 100 000-person target population were calculated for each PrEP strategy, assuming each person received PrEP.

The 5 prevention strategies evaluated were (1) no PrEP, (2) continuous CAB-LA ($22 200 pppy), (3) continuous CAB-LA at target price ($250 pppy), (4) continuous TDF/FTC ($48 pppy), and (5) event-driven TDF/FTC ($12 pppy). Assumed efficacy was 90% (continuous CAB-LA) and 60% (continuous and event-driven TDF/FTC). Thirty percent efficacy was used for a sensitivity analysis on event-driven TDF/FTC.

Annual costs for continuous CAB-LA and continuous TDF/FTC are based on currently available branded, target and generic prices, respectively [19, 20]. Annual event-driven TDF/FTC is estimated at $12 pppyassuming 3 months of event-driven PrEP use pppy, based on existing usage estimates [16].

For each PrEP strategy, there was an assumed additional cost of $4 pppy for HIV testing and $14 pppy for education and service access, based on existing cost estimates [16]. Therefore, the final costs used are $22 218 (current-price CAB-LA), $268 (target-price CAB-LA), $66 (continuous oral TDF/FTC), and $30 (event-driven oral TDF/FTC).

Efficacy estimate justifications are in Supplementary Appendix 5. Based on increasing evidence that continuous and event-driven TDF/FTC efficacy is comparable, 60% efficacy was used for both strategies [15, 17, 18]. Half of the estimated continuous TDF/FTC efficacy was used for event-driven sensitivity analysis.

For each PrEP strategy, NNTB, cost of treating the entire target population, and cost per HIV infection prevented were also calculated using the aforementioned costs.

NNTB was calculated using the standard formula:


1/(KE),where

*K* is the weighted mean incidence per 100 PY of a given key population divided by 100 and *E* is the estimated percentage efficacy of a given PrEP strategy divided by 100.

For example, the CAB-LA NNTB of a population with a 2.5 incidence would be calculated as follows:


K=0.025

E=0.9

1/(0.9×0.025)=44.4

NNTB=44.4

**Figure 1.**
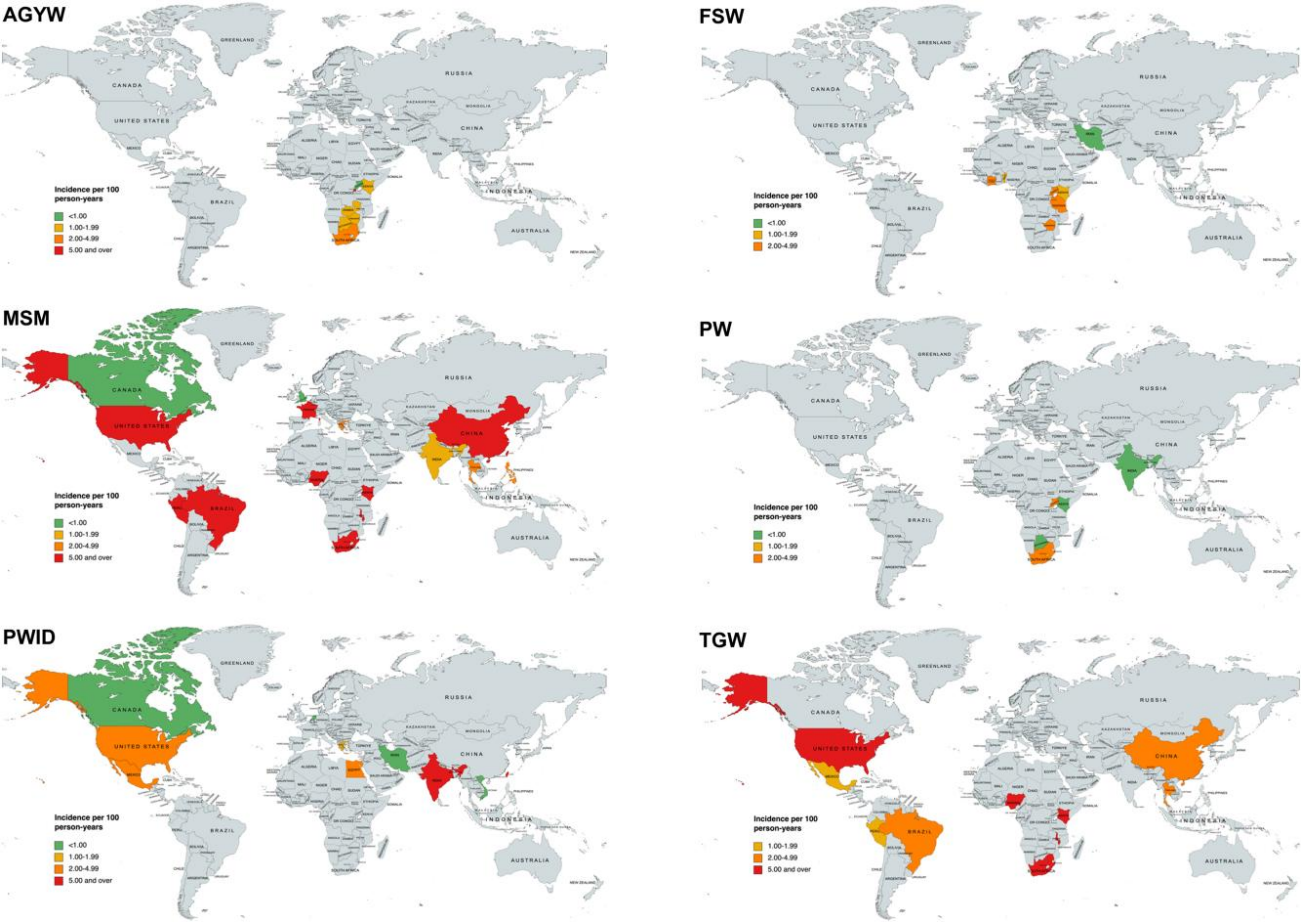
Human immunodeficiency virus incidence for adolescent girls and young women (AGYW; mean incidence, 2.08 per 100 person-years [PY]; number of studies, 20); female sex workers (FSWs; mean incidence, 3.10 per 100 PY; number of studies, 7); men who have sex with men (MSM; mean incidence, 2.62 per 100 PY; number of studies, 31); pregnant women (PW; mean incidence, 0.88 per 100 PY; number of studies, 9); people who inject drugs (PWID; mean incidence, 3.82 per 100 PY; number of studies, 12); and transgender women (TGW; mean incidence, 3.17 per 100 PY; number of studies, 10).

**Figure 2.**
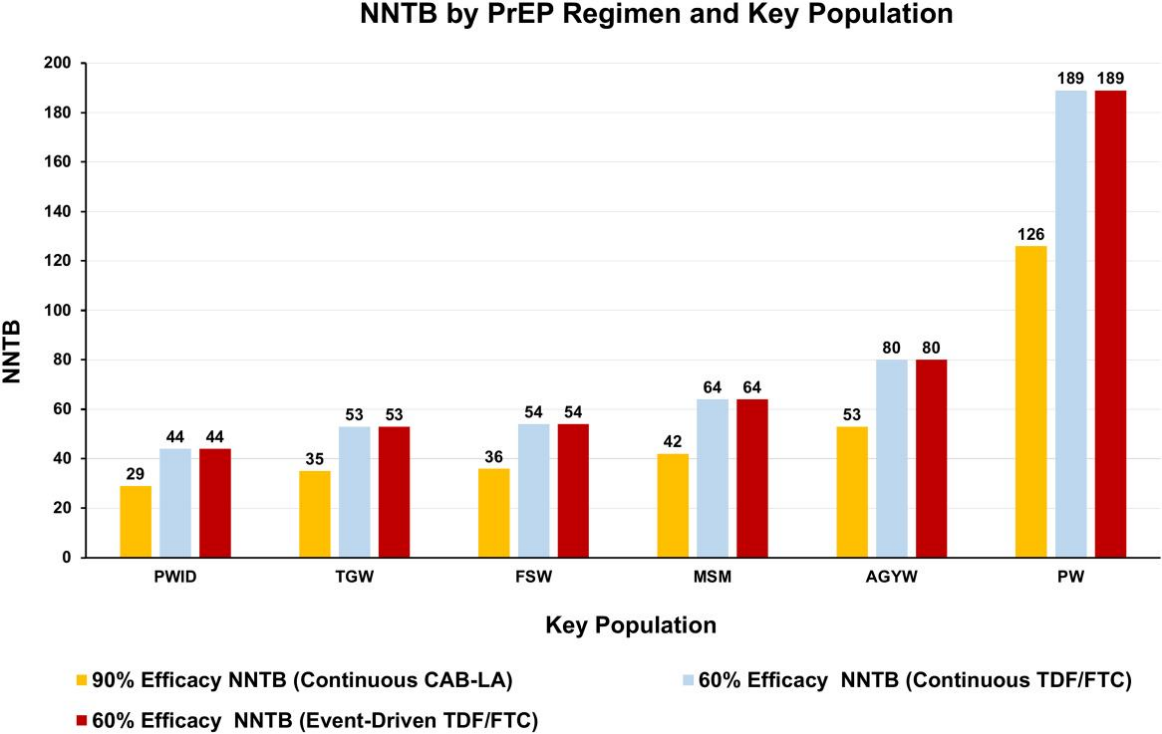
Number needed to treat to benefit (NNTB) for each preexposure prophylaxis (PrEP) medication (long-acting cabotegravir [CAB-LA], tenofovir disoproxil fumarate/emtricitabine [TDF/FTC]) by key population. For event-driven sensitivity analysis, the NNTB was 87 for people who inject drugs (PWID), 105 for transgender women (TGW), 108 for female sex workers (FSWs), 127 for men who have sex with men (MSM), 160 for adolescent girls and young women (AGYW), and 378 for pregnant women (PW).

Given incidence, the NNTB is lowest in the most efficacious therapy, CAB-LA. Weighted mean incidence and NNTB values are shown in Figures 1 and 2, respectively.

Cost per infection prevented was calculated by multiplying NNTB by the price pppy of a given PrEP strategy.

## RESULTS

### Global Incidence

Ninety-eight studies in 5 230 189 individuals were included for analysis. All included studies were appraised with either moderate or high study quality.

Mean incidence per 100 PY was 0.03 in blood donors, 0.88 in PW, 1.06 in the general population, 2.08 in AGYW, 2.62 in MSM, 3.10 in FSWs, 3.17 in TGW, and 3.82 in PWID. The full results are shown in Supplementary Appendix 6. Global HIV incidence by key population is shown in Figure 1.

Of the at-risk populations, PW had the lowest incidence. All other at-risk populations had higher average HIV incidence than the general population. Mean blood donor incidence was much lower than the general population, due to strict screening processes for blood-borne viruses including HIV.

Of 14 studies in the general population that were stratified either by sex or single sex, mean incidence was 5.3 times higher in women compared to men.

There were higher estimates of HIV incidence in Southern Africa and East Africa, compared with other regions, as shown in Figure 1. Full results for Southern Africa and East Africa are shown in Supplementary Appendix 7.

### Implications for Affordability

Mean HIV incidence across the at-risk populations was 2.6 per 100 PY (MSM, TGW, FSWs, PWID, PW, and AGYW excluding blood donors and excluding the general population). Using this, NNTB was calculated for each PrEP strategy: 42.7 (CAB-LA), 64.1 (continuous and event-driven TDF/FTC), and 128.2 (sensitivity analysis). Assuming 100 000 people in at-risk populations receive PrEP, Table 3 shows, by PrEP strategy, the cost of treating the entire 100 000 target population, the NNTB, the mean number of HIV infections remaining in the target population, and the mean cost per infection prevented. Figure 2 shows NNTB by risk population for each strategy.

**Table 3.** Cost to Treat a 100 000-Target Population, Number Needed to Treat to Benefit, Human Immunodeficiency Virus Infections Remaining in the Target Population, and Cost per Infection Prevented by Preexposure Prophylaxis Strategy

Type of PrEP	Cost to Treat 100 000 Target Population, US$	NNTB	HIV Infections, No.	Cost per Infection Prevented, US$
No PrEP	0	NA	2600	0
TDF/FTC (continuous)	6 600 000	64.1	1040	4231
TDF/FTC (event-driven)	3 000 000	64.1	1040	1923
CAB-LA (US price)	2 221 800 000	42.7	260	949 487
CAB-LA (target price)	26 800 000	42.7	260	11 453

Mean incidence: 2.6 per 100 person-years.

Abbreviations: CAB-LA, long-acting cabotegravir; HIV, human immunodeficiency virus; NA, not applicable; NNTB, number needed to treat to benefit; PrEP, preexposure prophylaxis; TDF/FTC, tenofovir disoproxil fumarate/emtricitabine; US$, United States dollars.

For example, in 100 000 MSM in Brazil (incidence of 6.65 per 100 PY; Figure 1), using no PrEP would result in 6650 new HIV infections. Treating the same population with current-price CAB-LA, with a 16.7 NNTB, 5985 infections can be prevented, leaving 665 infections remaining, costing $2 221 800 000 to treat all 100 000, and $371 228 per infection prevented. With event-driven TDF/FTC (at 60% efficacy) with a 25.1 NNTB, 3990 infections can be prevented, leaving 2660 infections remaining, costing $3 000 000 to treat all 100 000, and $752 per infection prevented.

Even at 30% efficacy for event-driven sensitivity analysis and a 50.1 NNTB, 1995 infections can be prevented, leaving 4655 infections remaining, costing $3 000 000 to treat all 100 000, and $1504 per infection prevented.

**Figure 3.**
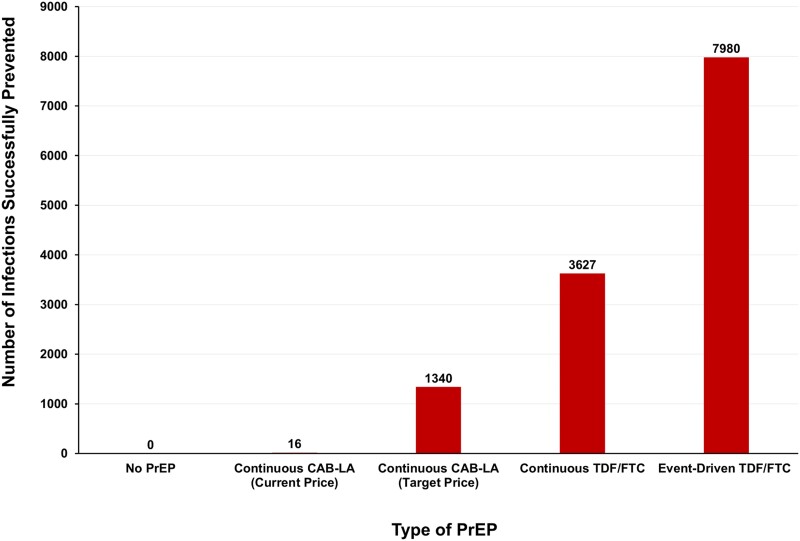
The number of infections successfully prevented in men who have sex with men in Brazil within a $6 million budget. Abbreviations: CAB-LA, long-acting cabotegravir; PrEP, preexposure prophylaxis; TDF/FTC, tenofovir disoproxil fumarate/emtricitabine.

Considering Brazil's $6 million estimated PrEP budget [30], using cost per infection prevented, CAB-LA would successfully prevent 16 infections, whereas event-driven TDF/FTC could prevent 7980 infections in MSM. Event-driven sensitivity analysis could successfully prevent 3990 infections. Full results are shown in Figure 3.

This trend was seen across all populations; the cost per infection prevented is much higher in CAB-LA than event-driven TDF/FTC, despite the lower NNTB.

## DISCUSSION

Incidence per 100 PY ranged from 0.03 in blood donors to 3.82 in PWID. The same trends were seen across all populations: Event-driven TDF/FTC costs the least per infection prevented, resulting in more successfully prevented infections within fixed budgets, despite lower NNTBs.

Compared to existing literature, this review provides a comprehensive analysis of the impact, on multiple risk populations, of 3 different PrEP strategies at 4 price points, versus no PrEP, given a limited PrEP budget. Studies were published in 2018 onward, and PrEP costing, budgets, and efficacies used were up to date [31]. This ensures that recent HIV data contribute to the analysis, reflecting the status of HIV, considering influences of recent world events, for example, the coronavirus disease 2019 (COVID-19) pandemic. Existing literature on cost-effectiveness of event-driven TDF/FTC and unrealistic pricing of CAB-LA support this review. IPERGAY, PREVINIR, and cost-effectiveness studies on event-driven PrEP demonstrate that it is equally as efficacious as continuous PrEP [15–18]. Investigations including a CAB-LA cost-effectiveness analysis by Jamieson et al criticize its inaccessibility and impracticality, calling for marked reductions in its cost to be an effective and realistic PrEP option [11, 19, 20, 32].

Limitations of this study should be noted. Despite a thorough search strategy, most studies screened from 2018 onward did not report incidence; most reported prevalence. This is potentially due to follow-up difficulties, particularly considering COVID-19. Where incidence was reported, this was mostly in randomized controlled trials measuring preventive interventions, or in mathematical modeling studies to estimate projected incidence. These were excluded given concerns regarding external validity and variability in model parameters, respectively [33]. This limited the amount of incidence data included and the geographical areas covered within the analysis, potentially introducing selection bias and reducing accuracy of incidence estimates. Furthermore, efficacy estimates for all 4 drug-based PrEP strategies were difficult to determine directly, due to ranging efficacies reported in existing studies. However, the use of multiple trial results, conducted in multiple risk groups in each estimation, increases the representativeness and reliability of the estimates.

Based on NNTB, the average cost per HIV infection prevented using CAB-LA is $949 487 across at-risk populations, surpassing the total annual spending for many countries (Table 2).

Considering opportunity cost, wide-scale CAB-LA usage would significantly reduce numbers of infections successfully prevented and money available for other preventive services [34]. Furthermore, widespread CAB-LA use incurs additional time and financial costs, as administering injections requires additional appointment time, training, and staff.

As regards Brazil, this country has been excluded from the ViiV-MPP voluntary license. If Brazil had to pay high commercial prices for CAB-LA, then it would be better to use generic TDF/FTC as the main PrEP strategy, as more HIV infections could be prevented (Figure 3). An alternative used previously in Brazil is compulsory licensing. If the CAB-LA patent was revoked, then generic low-cost supplies could be imported; this is a legal mechanism to gain access under the World Trade Organisation's Agreement on Trade-Related Aspects of Intellectual Property Rights 1995 (TRIPS). This international legislation permits compulsory licensing to promote better access to medicines, and to improve research and development into new medications.

Predicting CAB-LA costing and availability changes is difficult, given increasing pressure to lower prices [11, 20]. Its viability is dependent on cost reduction: Jamieson et al suggest that CAB-LA could be a cost-effective solution, if the price were $63.21–$101.29 pppy [32]. Given its estimated generic cost of $16–$34 pppy, this could be possible [11].

However, the lack of CAB-LA approval in low- and middle- income countries, and limited research on CAB-LA effects including during pregnancy or coinfection, means that it is unlikely to be widely available in the immediate future [35].

Furthermore, HIV reduction is possible without widespread CAB-LA use, as seen in countries including Australia, where significant HIV incidence declines, owing to regular testing and PrEP use, have been achieved [36]. While differing cultural and sociopolitical contexts influence these behaviors, this demonstrates that CAB-LA may not be a necessity for global HIV prevention.

Therefore, event-driven TDF/FTC should be used where possible, to maximize prevention within budget. PrEP spending variability (Table 2) highlights the nuance in allocating finances where demands for HIV and competing healthcare resources are high and budgets are low. Therefore, pushes for increased generic manufacture, PrEP availability, and global awareness of the benefits of prioritizing and de-stigmatizing PrEP use in at-risk populations would be valuable.

This analysis favored low-cost, event-driven TDF/FTC ($30 pppy), both at 60% and 30% efficacies, for use among at-risk groups. Event-driven TDF/FTC has growing evidence that it has comparable efficacy to continuous TDF/FTC, thus is a strong candidate for widespread HIV prevention [15, 17, 18]. This warrants further investigation into event-driven TDF/FTC effectiveness, implementation, and adherence in more populations, since existing studies are concentrated in MSM [17, 18].

This includes observing event-driven TDF/FTC effectiveness, using different settings and distribution methods. A PrEP cost-effectiveness analysis conducted by Phillips et al discussed using comprehensive event-driven “prevention packages” including service access, HIV and health education, sexually transmitted infection self-test kits, and condoms, alongside PrEP [16].

Event-driven TDF/FTC distribution could be through “Protection Packs” in schools, for AGYW. Contents would be like prevention packages, with additional information, advice, and GBV support signposting. Labeling them as “protection” would better reflect their purpose, steering away from common misconceptions about PrEP and promiscuity [8–10]. This could help to tackle high AGYW incidence, given the GBV risk. It presents opportunities to address pervasive stigma and lack of awareness surrounding HIV and PrEP use, by educating AGYW, their parents, and the wider school community, and to reduce “PrEP shaming” through normalization of PrEP ownership.

Moreover, the $12 cost of event-driven TDF/FTC (medication only) was modeled on 3 months’ usage pppy [11, 16]. Instead, by providing 1 month's supply ($4 pppy) with contact details to obtain more if required, packs can be given to most adolescents, but money lost and medicines wasted from disuse would be minimized. Providing contacts to at-risk adolescents also presents an opportunity to engage them with medical care and support.

Phillips et al also discuss the benefits of providing readily available postexposure prophylaxis alongside event-driven PrEP, for instances where HIV exposure is less predictable [16]. Further analysis should measure the effectiveness of this addition to the packs, particularly for AGYW.

The World Health Organization recommendations also support TDF/lamivudine (3TC) use where TDF/FTC availability is limited [37]. TDF/3TC can also be event-driven, and future research should compare its effectiveness and affordability with other PrEP regimens.

Acceptability of event-driven PrEP strategies, particularly considering “PrEP shaming,” should also be investigated. Establishing practical and social implications surrounding event-driven PrEP use reduces risk of low adherence despite high efficacy, as with oral daily TDF/FTC [38]. Similarly, evidence should be collected in at-risk populations, in the community, on implementation and acceptability of CAB-LA, for comparison between event-driven versus continuous PrEP [39, 40].

Alongside those in this study, event-driven PrEP could benefit other at-risk groups, particularly those with poor healthcare access or engagement. Research measuring incidence in difficult-to-follow groups should be conducted, to understand their needs for preventive service provision.

## CONCLUSIONS

CAB-LA is the most efficacious form of PrEP, but high prices limit the number of people who can be treated. More HIV infections can be prevented using low-cost, event-driven TDF/FTC as PrEP, as far more people can be treated within fixed budgets. This result was consistent across a range of at-risk populations in different countries. Therefore, event-driven PrEP should be further investigated for HIV prevention, as the current CAB-LA pricing is unrealistic for population-level treatment.

## Supplementary Data

Supplementary materials are available at *Clinical Infectious Diseases* online. Consisting of data provided by the authors to benefit the reader, the posted materials are not copyedited and are the sole responsibility of the authors, so questions or comments should be addressed to the corresponding author.

## Supplementary Material

ciad537_Supplementary_Data

## Notes

***Author contributions.*** Both authors contributed equally to this work.

***Financial support.*** This research is supported by the International Treatment Preparedness Coalition (ITPC) Make Medicines Affordable Campaign (grant number ITPC/MV/2021 to I. S. and A. H.).
