# Supplementary material for: Global HIV Incidence Analysis and Implications for Affordability Using Long-Acting Cabotegravir Versus Continuous and Event-Driven Oral Preexposure Prophylaxis
Source: Clin Infect Dis. 2023 Sep 4;78(2):386–94. doi: 10.1093/cid/ciad537 (PMC10874262; doi:10.1093/cid/ciad537)
Supplement: ciad537_Supplementary_Data [file ciad537_supplementary_data.docx]

**Appendices**

**Appendix 1: Search Strategy**

**
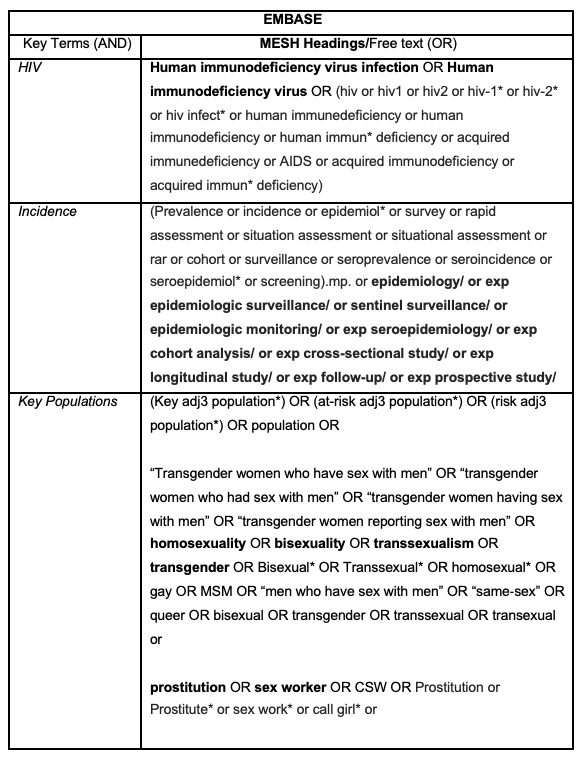
**

**
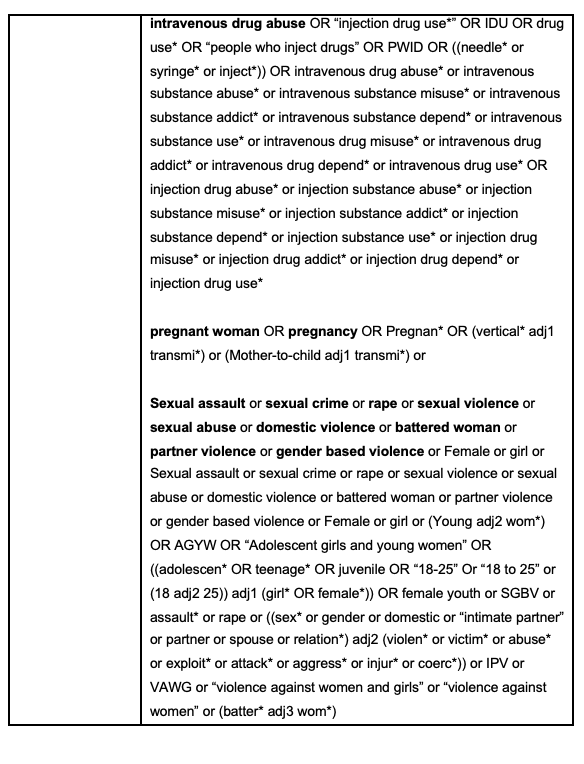
**


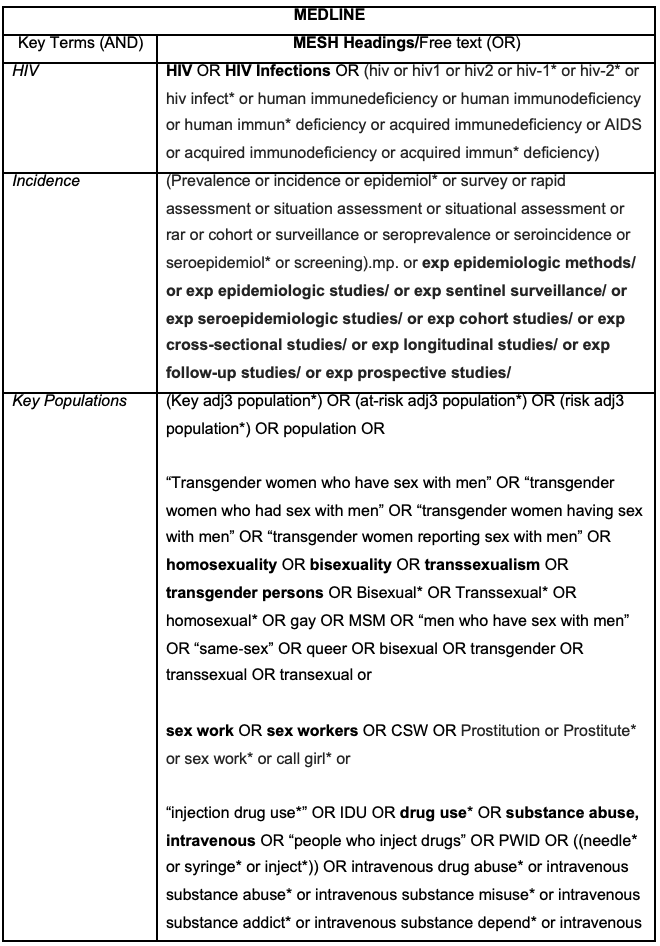


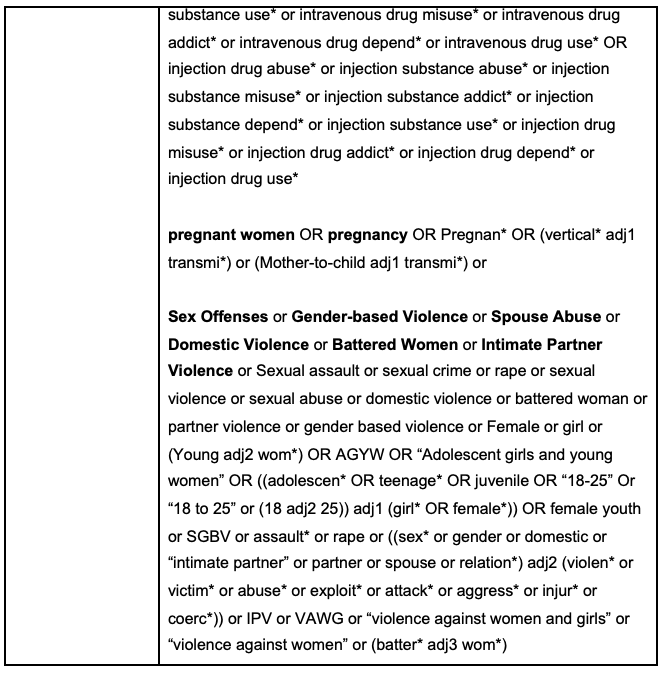


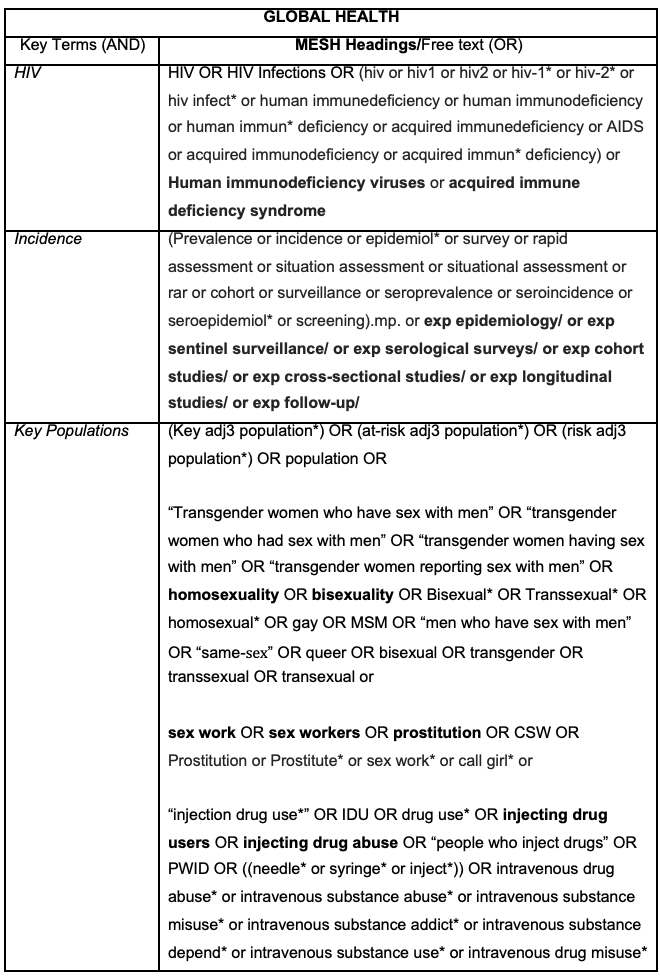

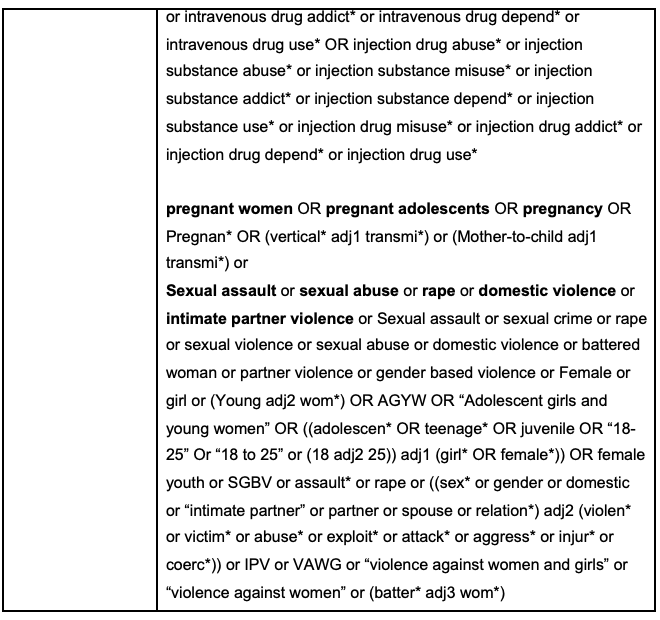


**Appendix 2: PICOS Criteria**

***Table: Table summarising PICOS inclusion and exclusion criteria used to determine study eligibility.***

| **Parameter** | **Inclusion** | **Exclusion** |
| --- | --- | --- |
| *Population* | HIV-epidemic affected/at-risk key populations including:   - Men/transgender women who have sex with men (MSM, TGW) - Pregnant Women (PW) - People who inject drugs (PWID) - Commercial Sex Workers (CSW) - Adolescent Girls and Young Women (AGYW)   Countrywide or general populations  Blood Donors | Specific subsets of the population which have not been defined in the inclusion criteria, e.g partners of participants in key/at-risk populations  Paediatric populations (defined as under 13 years of age) |
| *Intervention* | N/A (Epidemiological Studies) | N/A |
| *Comparator* | N/A (Epidemiological Studies) | N/A |
| *Outcomes* | Incidence or seroconversion (rate of new cases/occurrences). | Prevalence only  Any studies not including a measure for incidence or seroconversion rate |
| *Study* | Epidemiological/sero-epidemiological studies measuring incidence (including cohort, cross-sectional, surveillance, longitudinal, follow up, prospective studies)  Studies/clinical trials conducted in humans | Animal/non-human studies  Studies not measuring HIV incidence in populations of interest  Qualitative Studies  Randomised controlled trials comparing PrEP interventions  Studies modelling/predicting incidence |

**Appendix 3: Data Extraction Table**

***Table: Data extraction table summarising included studies, data collected for parameters of interest, and quality appraisal for each study.***

****Some studies had data unavailable or missing for ‘Years of Study’, ‘Number infected’, and ‘HIV Antibody Type Test’ parameters. These missing fields have been demarcated with an asterisk (*).***

| **Study** | **Sample Size** | **Country** | **Risk Population** | **Incidence per 100pys** | **Years of Study** | **Number infected** | **HIV Antibody Test Type** | **Quality Appraisal (High/Moderate/Low)** |
| --- | --- | --- | --- | --- | --- | --- | --- | --- |
| Borgdorff, M W. et al^41^ | 1934 | Kenya | AGYW 15-24 years; | 0.89 | 2012-2016 | 51 | Rapid Test | Moderate (7) |
|  | 4586 | Kenya | 15-64 years men; | 0.64 | 2012-2016 | 94 | Rapid Test | Moderate (7) |
| Kasamba, I. et al^42^ | 2206 | Uganda | CSW; | 3.07 | 2008-2017 | 170 | Rapid Test | Moderate (7) |
| Kimani, M. et al^43^ | 42 | Kenya | MSM; | 4.5 | 2016-2017 | 2 | * | Moderate (6) |
|  | 14 | Kenya | TGW; | 20.6 | 2016-2017 | 3 | * | Moderate (6) |
| Lane, T. et al^44^ | 179 | South Africa | MSM; | 12.5 | 2012-2015 | 18 | Rapid Test | Moderate (6) |
| Li, Q. et al^45^ | 523 | China | MSM; | 6.6 | 2014-2015 | 16 | ELISA | Moderate (6) |
| Mi, G. D. et al^46^ | 1937 | China | MSM; | 3.47 | 2017-2018 | 37 | Rapid Test | Moderate (8) |
| Scheim, A. I. et al^47^ | 1131 | Canada | PWID; | 1.26 | 1996–2014 | 102 | * | Moderate (7) |
| Sharifi, H. et al^48^ | 1339 | Iran | CSW | 0.112 | 2015 | 27 | ELISA and Rapid Tests | Moderate (8) |
|  | 2092 | Iran | PWID; | 0.539 | 2014 | 213 | 2 ELISAs | Moderate (8) |
| Vandormael, A. et al^49^ | 12609 | South Africa | Women | 3.06 | 2017 | 92 | ELISA | High (10) |
|  | 9630 | South Africa | Men | 1.01 | 2017 | 20 | ELISA | High (10) |
| de Oliveira Garcia Mateos, S. et al^50^ | 326177 | Brazil | First Time Blood Donors Recife | 0.0451 | 2007-2016 | * | Recency test | Moderate (6) |
|  | 230259 | Brazil | First Time Blood Donors Belo Horizonte | 0.0233 | 2007-2016 | * | Recency test | Moderate (6) |
|  | 373744 | Brazil | First Time Blood Donors Sao Paulo | 0.0322 | 2007-2016 | * | Recency test | Moderate (6) |
|  | 585249 | Brazil | Repeat Blood Donors Recife | 0.0332 | 2007-2016 | * | Chemiluminescence Assay | Moderate (6) |
|  | 330413 | Brazil | Repeat Blood Donors Belo Horizonte | 0.0275 | 2007-2016 | * | Chemiluminescence Assay | Moderate (6) |
|  | 698510 | Brazil | Repeat Blood Donors Sao Paulo | 0.017 | 2007-2016 | * | Chemiluminescence Assay | Moderate (6) |
| Schumann, H. et al^51^ | 1610 | Uganda | Pregnant Women; | 2.85 | 2017 | 15 | Rapid Test | Moderate (6) |
| Moyo, S. et al^52^ | 2,757 | Botswana | 16–49-year-olds; | 1.29 | 2013-2015 | 34 | Recency Test | Moderate (7) |
| Psomas, C. K. et al^53^ | 84 | France | MSM; | 5.95 | 2016-2018 | 10 | * | Moderate (6) |
| Mushamiri, I. et al^54^ | 7379 | Eswatini | 18–49-year-olds; | 2.4 | 2010-2015 | 145 | Rapid Test | High (9) |
| Schaefer, R. et al^55^ | 3746 | Zimbabwe | AGYW 15-24 years; | 1.43 | 1998/2013 | 126 | * | Moderate (7) |
| Donnell, D. et al^56^ | 82 082 | England | MSM; | 0.28 | 2018 | 153 | * | Moderate (6) |
| Anwar, S. et al^57^ | 240 | Egypt | PWID; | 3.9 | 2013-2017 | 5 | * | Moderate (7) |
| Albert, L. M. et al^58^ | 2317 | South Africa | AGYW; | 2.17 | 2011-2017 | 197 | 2 rapid tests and HIV-1 western blot assay | High (9) |
| LeeVan, E. et al^59^ | 138 | Nigeria | TGW; | 13.1 | 2013-2020 | * | Rapid Test | Moderate (7) |
|  | 1338 | Nigeria | MSM; | 8.7 | 2013-2020 | * | Rapid Test | Moderate (7) |
| Amelia de Sousa Mascena Veras, M. et al^60^ | 545 | Brazil | TGW; | 2.68 | 2017-2019 | 13 | * | Moderate (5) |
| Javanbakht, M. et al^61^ | 102 | USA | MSM; | 5.9 | 2014-2020 | 6 | * | Moderate (5) |
|  | 115 | USA | MSM; | 4.3 | 2014-2020 | 5 | * | Moderate (5) |
| Nyabuti, M. N. et al^62^ | 52286 | Uganda, Kenya | Whole male population; | 0.22 | * | 261 | Bio-Rad Genius HIV 1/2 assay/Western blot testing | Moderate (7) |
|  | 42453 | Uganda, Kenya | AGYW 15-24 years; | 0.4 | * | 176 | Bio-Rad Genius HIV 1/2 assay/Western blot testing | Moderate (7) |
| Chimbindi, N. et al^63^ | 2482 | South Africa | AGYW 20-24 years | 7.45 | 2011‒2016 | 289 | Dried Blood Spot Testing | High (10) |
|  | 3536 | South Africa | AGYW 15-19 years | 4.54 | 2011‒2015 | 197 | Dried Blood Spot Testing | High (10) |
| Ravindran, J. et al^64^ | 1244 | Kenya | Pregnant Women; | 0.8 | 2009-2015 | 10 | Nucleic Acid Amplification Test | Moderate (6) |
| Bhushan, N. L. et al^65^ | 2245 | South Africa | AGYW 13–20 years; | 1.94 | 2011/2017 | 174 | Rapid Test | Moderate (5) |
| Nanthaprut, P. et al^66^ | 6119 | Thailand | MSM; | 3.7 | 2017-2019 | 224 | Rapid Test | Moderate (7) |
|  | 825 | Thailand | TGW; | 5.5 | 2017-2020 | 45 | Rapid Test | Moderate (7) |
| van Santen, D. K. et al^67^ | 624 | The Netherlands | PWID; | 0.74 | 1985–2013 | 57 | * | Moderate (7) |
|  | 1399 | Canada | PWID; | 0.34 | 1997-2009 | 37 | * | Moderate (7) |
| Samudyatha, U. C. et al^68^ | 2500 | India | Pregnant Women; | 0.118 | 2018-2019 | 2 | Rapid Test | Moderate (6) |
| Knox, J. et al^69^ | 416 | USA | MSM; | 5.3 | 2012-2014 | 22 | Rapid Test | Moderate (7) |
|  | 33 | USA | TGW; | 6.1 | 2012-2014 | 2 | Rapid Test | Moderate (7) |
| Clipman, S. J. et al^70^ | 782 | India | PWID; | 21.3 | 2017-2020 | 159 | * | Moderate (8) |
| Goin, D. E. et al^71^ | 2,415 | South Africa | AGYW 13–21 years; | 0.072 | 2011-2017 | 175 | Rapid Test | Moderate (8) |
| Willcox, A. C. et al^72^ | 670 | Kenya | CSW; | 1.36 | 2010-2017 | 17 | ELISA | Moderate (7) |
| Diabaté, S. et al^73^ | 188 | Benin | CSW; | 1.41 | 2008-2012 | 6 | Rapid Test | Moderate (5) |
| Wongjarupong, N. et al^74^ | 43,084 | Burkina Faso | Blood donors; | 1.121 | 2009-2013 | 2,106 | Vironostika HIV Uni-Form II Ag/Ab | High (9) |
| Negedu-Momoh, O. R. et al^75^ | 127 | Nigeria | whole male population; | 0.42 | 2017 | * | Recency test | Moderate (6) |
|  | 243 | Nigeria | whole female population; | 0.41 | 2017 | * | Recency test | Moderate (6) |
| Birdthistle, I. et al^76^ | 6528 | South Africa | AGYW - 15 to 24 years; | 4.07 | 2016-2018 | 249 | ELISA | High (10) |
| Teixeira, S.L. et al^77^ | 2429 | Brazil | MSM; | 6.65 | 2018-2020 | 65 | Recency test | Moderate (6) |
|  | 458 | Brazil | TGW; | 9.16 | 2018-2021 | 8 | Recency test | Moderate (6) |
| Konda, K. A. et al^78^ | 494 | Brazil, Mexico, Peru | TGW; | 1.82 | 2018-2021 | 5 | Rapid Test | Moderate (5) |
| Hui, S. et al^79^ | 3,512 | China | MSM; | 3.55 | 2013-2018 | 151 | ELISA or Rapid test, Western blotting for confirmation | Moderate (5) |
| Mutisya, E. M. et al^80^ | 174 | Kenya | MSM; | 4.58 | 2015-2017 | 8 | Rapid Test | Moderate (7) |
| Sturt, A. S. et al^81^ | 492 | Zambia | AGYW 18-31 Women; | 1.72 | 2018 | 20 | Laboratory-based fourth-generation HIV-1 testing | Moderate (6) |
| Samji, H. et al^82^ | 9038 | Canada | MSM; | 0.85 | 2004-2017 | 257 | * | Moderate (7) |
| Tunnage, J. et al^83^ | 619 | Kenya | Whole population 18-35 years; | 2.74 | 2017-2018 | 24 | Rapid Test or ELISA | Moderate (8) |
| Skaathun, B. et al^84^ | 414 | USA/Mexico | PWID; | 5.15 | 2020-2021 | 9 | * | Moderate (6) |
| Palanee-Phillips, T. et al^85^ | 5768 | South Africa | AGYW 16–35 years | 4.51 | 2015-2018 | 345 | * | Moderate (8) |
| Ssempijja, V. et al^86^ | 8056 | Uganda | 15-49-year-old men | 0.42 | 2013-2018 | 60 | Rapid Test | Moderate (7) |
|  | 9325 | Uganda | 15-49-year-olds women | 0.69 | 2013-2018 | 112 | Rapid Test | Moderate (7) |
| Ditangco, R. et al^87^ | 708 | Philippines | MSM; | 2.7 | 2014-2018 | 56 | HIV antibody test (HAT) and PCR test | Moderate (8) |
| Lewis, L. et al^88^ | 2,710 | South Africa | AGYW (15–24 years); | 3.92 | 2014-2017 | 163 | * | High (9) |
| Lambert, G. et al^89^ | 2,008 | Canada | MSM; | 0.4 | 2017-2021 | 31 | Fourth-generation HIV testing ad Western Blot | Moderate (8) |
| Shan, D. et al^90^ | 439 | China | TGW; | 4.42 | 2016-2018 | 23 | Rapid Test | Moderate (8) |
|  | 617 | China | MSM; | 1.35 | 2016-2018 | 10 | Rapid Test | Moderate (8) |
| Sandfort, T. G. M. et al^91^ | 271 | Kenya; Malawi; South Africa | MSM; | 6.8 | 2015–2017 | 17 | Rapid Test | Moderate (7) |
|  | 53 | Kenya; Malawi; South Africa | TGW; | 8.4 | 2015–2017 | 4 | Rapid Test | Moderate (7) |
| Ussery, F. et al^92^ | 2681 | Botswana | AGYW 16-24; | 1.87 | 2013-2018 | 66 | Rapid Test and ELISA | Moderate (7) |
|  | 13018 | Botswana | 16-64 men | 0.34 | 2013-2018 | 42 | Rapid Test and ELISA | Moderate (7) |
| Nkambule, R. et al^93^ | 1300 | Eswatini | AGYW 18-25-year-old women; | 1.7 | 2016 | 7 | Rapid Test | Moderate (8) |
| Phanuphak, N. et al^94^ | 13511 | Thailand | MSM; | 0.459 | 2018 | 55 | Recency Test | Moderate (7) |
| Li, M. et al^95^ | 1043 | China | MSM; | 16 | 2017-2018 | 98 | Rapid Test | Moderate (6) |
| Steele, W. R. et al^96^ | 932148 | USA | Blood donors; | 0.00258 | 2015-2019 | 18 | * | Moderate (7) |
|  | 838 473 | USA | Blood donors; | 0.00038 | 2015-2019 | 2 | * | Moderate (7) |
| Luz, E. et al^97^ | 135,490 | Brazil | Blood donors aged 16-69 years; | 0.0543 | 2008-2017 | 456 | Enzyme immunoassay and/or Chemiluminescence | Moderate (8) |
| Björkman Nyqvist, M. et al^98^ | 535 | Lesotho | 18–32-year-olds; | 12.9 | 2010-2012 | 69 | Rapid Test | Moderate (7) |
| Penumetsa, M. et al^99^ | 5,878 | Kenya | Pregnant Women; | 0.5 | 2017-2019 | 18 | Fourth-generation HIV assay | Moderate (7) |
| Nouaman, M. N. et al^100^ | 1000 | Côte d’Ivoire | CSW; | 2.3 | 2016–2017 | 39 | Rapid Test and Dried Blood Spot | Moderate (6) |
| Mussa, A. et al^101^ | 86,282 | Botswana | Pregnant Women; | 0.69 | 2017-2021 | 223 | Rapid Test | High (9) |
| Hoffman, S. et al^102^ | 1771 | Uganda | 15–24-year-old Men; | 0.977 | 2005-2013 | 48 |  | Moderate (6) |
| Abrahams, N. et al^103^ | 441 | South Africa | 16–40 years women exposed to rape; | 6.6 | 2014-April 2019 | 37 | Rapid Test and ELISA | High (9) |
|  | 578 | South Africa | 16–40 years women (not exposed to rape); | 4.7 | 2014-2019 | 49 | Rapid Test and ELISA | High (9) |
| Jones, H. S. et al^104^ | 7573 | Zimbabwe | CSW; | 3.9 | 2009-2019 | 464 | Rapid Test | Moderate (8) |
| Jain, J.P. et al^105^ | 651 | Mexico | PWID; | 1.25 | 2011-2018 | 43 | Rapid Test | High (9) |
| Kilburn, K. et al^106^ | 2362 | South Africa | AGYW 13–20 years old; | 2 | 2011-2012 | 107 | Rapid Test | Moderate (7) |
| Kritsanavarin, U. et al^107^ | 686 | Thailand | MSM; | 6.42 | 2015–2018 | 33 | Rapid Test | Moderate (7) |
| Akullian, A. et al^108^ | 9801 | South Africa | 15 to 54 y old among men; | 2 | 2004-2019 | 887 | ELISA | High (9) |
|  | 12605 | South Africa | 15 to 49 y old among women | 4.5 | 2004-2019 | 2687 | ELISA | High (9) |
|  | 3318 | South Africa | AGYW 15-19 | 3 | 2016-2019 | * | ELISA | High (9) |
|  | 3973 | South Africa | AGYW 20-24 | 5.12 | 2016-2019 | * | ELISA | High (9) |
| Machekano, R. et al^109^ | 850 | Lesotho | Pregnant Women; | 1.58 | 2013-2015 | 28 | Rapid Test | High (9) |
| Evans, K. N. et al^110^ | 857 | USA | PWID; | 0.93 | 2012-2019 | 22 | * | Moderate (5) |
| Nikolopoulos, G. K. et al^111^ | 1,243 | Greece | MSM; | 3.99 | 2013-2015 | 44 | Rapid Test | Moderate (5) |
| Lee, Y. C. et al^112^ | 27 | Taiwan | PWID; | 44.4 | 2006-2015 | 5 | Recency Testing | Moderate (8) |
|  | 15305 | Taiwan | MSM; | 12.2 | 2006-2015 | 776 | Recency Testing | Moderate (8) |
| Nowak, R. G. et al^113^ | 441 | Nigeria | MSM; | 15.4 | 2013-2018 | 81 | Rapid Test | Moderate (8) |
| Roussos, S. et al^114^ | 699 | Greece | PWID; | 1.94 | 2014-2020 | 57 | ELISA and Western blot | Moderate (8) |
| Piyaraj, P. et al^115^ | 1372 | Thailand | MSM; | 6 | 2006-2012 | 212 | Rapid Test | Moderate (7) |
| Celum, C. L. et al^116^ | 2,550 | Kenya, South Africa | AGYW 16–25-year-old girls and women; | 2.2 | 2017-2020 | 16 | Dried Blood Spot Test | High (9) |
| Hoque, M. et al^117^ | 798 | South Africa | Pregnant Women; | 17.3 | 2018 | 32 | Rapid test and ELISA | Moderate (8) |
| Woldesenbet, S. et al^118^ | 10,049 | South Africa | Pregnant Women; | 1.5 | 2017 | 136 | Recency Test | High (9) |
| Mohloanyane, T. et al^119^ | 76 | Lesotho | Males 10 years or older; | 5.26 | 2018-2019 | 2 | Recency Test | Moderate (6) |
|  | 108 | Lesotho | Females 10 years or older; | 20.4 | 2018-2019 | 11 | Recency Test | Moderate (6) |
| Rwibasira, G. et al^120^ | 2905 | Rwanda | Whole male population; | 5.09 | 2018–2020 | 148 | Recency Test | Moderate (8) |
|  | 1338 | Rwanda | AGYW (15–24 years); | 9.49 | 2018–2020 | 127 | Recency Test | Moderate (8) |
| Gras, J. et al^121^ | 203 | France and Canada | MSM; | 7.5 | 2012-2016 | 16 | ELISA | Moderate (7) |
| Mayer, M. E. et al^122^ | 1795 | Peru | MSM; | 11.9 | 2013-2015 | * | * | Moderate (8) |
| Yu, Z. et al^123^ | 2029 | China | MSM; | 3.36 | 2011–2019 | 127 | ELISA and Western blot | Moderate (7) |
| Rosa, P. B. et al^124^ | 118,374 | Brazil | 18-84 | 0.0276 | 2004-2019 | 708 | * | Moderate (7) |
| Justman, J. et al^125^ | 5746 | Eswatini (Swaziland) | Men 18-49; | 1.7 | 2011-2012 | 53 | Rapid Test | High (11) |
|  | 5486 | Eswatini (Swaziland) | Women 18-49; | 3.1 | 2011-2012 | 93 | Rapid Test | High (11) |
| Patel, E. U. et al^126^ | 10 003 | India | MSM; | 1.4 | 2016-2017 | 62 | Recency Test | Moderate (8) |
|  | 11 721 | India | PWID; | 5.2 | 2016-2017 | 248 | Recency Test | Moderate (8) |
| Reddy, T. et al^127^ | 188 | South Africa | AGYW (16–19 years); | 6.32 | 2012–2016 | * | * | High (9) |
|  | 897 | South Africa | AGYW (20-24 years); | 4.52 | 2012–2016 | * | * | High (9) |
| Mthiyane, N. et al^128^ | 1669 | South Africa | AGYW (13-22); | 2.2 | 2017-2019 | 52 | ELISA | High (10) |
|  | 1030 | South Africa | AGYW 13-22; | 2.2 | 2017-2018 | 52 | ELISA | High (10) |
| Moses, S. J. et al^129^ | 1407 | Liberia | Whole population; | 0.31 | 2015-2019 | 12 | Immunochromatographic assay | Moderate (6) |
|  | 1780 | Liberia | Whole population; | 0.34 | 2015-2019 | 17 | Immunochromatographic assay | Moderate (6) |
| Faini, D. et al^130^ | 700 | Tanzania | CSW; | 3.45 | 2018 | 21 | Rapid Test | Moderate (7) |
| Sudenga, S. L. et al^131^ | 1379 | USA, Mexico, Brazil | Men 18-70 years; | 0.276 | 2005-2015 | 29 | 4th Generation Antigen/Antibody Test | High (9) |
| Thienkrua, W. et al^132^ | 494 | Thailand | MSM; | 7.4 | 2006-2014 | 122 | Rapid Test | High (9) |
| Wand, H. et al^133^ | 9948 | South Africa | Women 16–49 years; | 7 | 2002–2016 | 856 | * | Moderate (8) |
| van Griensven, F. et al^134^ | 2943 | Thailand | TGW; | 1.03 | 2016-2019 | 12 | Chemiluminescence Assay | Moderate (8) |
| Laher, F. et al^135^ | 1249 | South Africa | 18-60 South Africa; | 3.1 | 2003-2020 | 45 | * | Moderate (6) |
| Des Jarlais, D. C. et al^136^ | 2569 | Vietnam | PWID; | 0.085 | 2016–2019 | 3 | Recency Test | Moderate (7) |
| Ortblad, K. F. et al^137^ | 29,570 | Botswana | Pregnant Women; | 0.8 | 2018-2019 | 17 | * | Moderate (8) |
| You, X. et al^138^ | 4578 | China | MSM; | 5.95 | 2012-2018 | 381 | * | Moderate (7) |

41.Borgdorff MW, Kwaro D, Obor D, Otieno G, Kamire V, Odongo F, Owuor P, Muthusi J, Mills LA, Joseph R, Schmitz ME, Young PW, Zielinski-Gutierrez E, De Cock KM. HIV incidence in western Kenya during scale-up of antiretroviral therapy and voluntary medical male circumcision: a population-based cohort analysis. *Lancet HIV*. 2018 May;5(5):e241-e249. doi: 10.1016/S2352-3018(18)30025-0. Epub 2018 Apr 9. PMID: 29650451.

42.Kasamba I, Nash S, Seeley J, Weiss HA. Human Immunodeficiency Virus Incidence Among Women at High-Risk of Human Immunodeficiency Virus Infection Attending a Dedicated Clinic in Kampala, Uganda: 2008-2017. *Sex Transm Dis*. 2019 Jun;46(6):407-415. doi: 10.1097/OLQ.0000000000000978. PMID: 31095103.

43.Kimani, M., van der Elst, E. M., Chiro, O., Oduor, C., Wahome, E., Kazungu, W., Shally, M., Rinke de Wit, T. F., Graham, S. M., Operario, D., & Sanders, E. J. (2019). PrEP interest and HIV-1 incidence among MSM and transgender women in coastal Kenya. *Journal of the International AIDS Society*, 22(6), e25323. <https://doi.org/10.1002/jia2.25323>

44.Lane, Tim PhD, MPH*; Osmand, Thomas MPH*; Marr, Alexander MPH*; Struthers, Helen PhD†; McIntyre, James A. MBChB, FRCOG†; Shade, Starley B. PhD*. Brief Report: High HIV Incidence in a South African Community of Men Who Have Sex With Men: Results From the Mpumalanga Men's Study, 2012–2015. *JAIDS Journal of Acquired Immune Deficiency Syndromes* 73(5):p 609-611, December 15, 2016. | DOI: 10.1097/QAI.0000000000001162

45.Li, Q., Li, X., Luo, Y., Fang, D., Chen, J., Zhang, X., Lv, X., Jin, J., Xu, K., Luo, W., & Qian, H. Z. (2019). HIV incidence and cohort retention among men who have sex with men in Hangzhou, China: A prospective cohort study*. Medicine*, 98(40), e17419. <https://doi.org/10.1097/MD.0000000000017419>

46.Mi, G. D., Zhang, B. B., Yu, F., Ren, X. L., Ong, J. J., Fan, Y. Q., Guo, F. H., Li, C. J., Zhang, M. Z., & Zhang, M. Y. (2021). HIV incidence among men who have sex with men using geosocial networking smartphone application in Beijing, China: an open cohort study. *Infectious diseases of poverty*, 10(1), 27. <https://doi.org/10.1186/s40249-021-00814-7>

47.Scheim, A. I., Nosova, E., Knight, R., Hayashi, K., & Kerr, T. (2018). HIV Incidence Among Men Who Have Sex with Men and Inject Drugs in a Canadian Setting. *AIDS and behavior*, 22(12), 3957–3961. <https://doi.org/10.1007/s10461-018-2185-3>

48.Sharifi, H., Mirzazadeh, A., Shokoohi, M., Karamouzian, M., Khajehkazemi, R., Navadeh, S., Fahimfar, N., Danesh, A., Osooli, M., McFarland, W., Gouya, M. M., & Haghdoost, A. A. (2018). Estimation of HIV incidence and its trend in three key populations in Iran. *PloS one*, *13*(11), e0207681. <https://doi.org/10.1371/journal.pone.0207681>

49.Vandormael, A. Akullian, M. Siedner, T. de Oliveira, T. Bärnighausen, F. Tanser, Declines in HIV incidence among men and women in a South African population-based cohort. *Nat. Commun*. 10, 5482 (2019).

50.de Oliveira Garcia Mateos S, Preiss L, Gonçalez TT, Di Lorenzo Oliveira C, Grebe E, Di Germanio C, Stone M, Amorim Filho L, Carneiro Proietti AB, Belisario AR, de Almeida-Neto C, Mendrone-Junior A, Loureiro P, Busch MP, Custer B, Cerdeira Sabino E; Recipient Epidemiology, Donor Evaluation Study (REDS-III) International Component Brazil. 10-year analysis of human immunodeficiency virus incidence in first-time and repeat donors in Brazil. *Vox Sang*. 2021 Feb;116(2):207-216. doi: 10.1111/vox.13002. Epub 2020 Sep 30. PMID: 32996602; PMCID: PMC8019535.

51.Schumann H, Rubagumya K, Rubaihayo J, Harms G, Wanyenze RK, Theuring S. The incidence of HIV and associated risk factors among pregnant women in Kabarole District, Uganda. *PLoS ONE*. 2020;15(6):e0234174. <https://doi.org/10.1371/journal.pone>.

52.Moyo S, Gaseitsiwe S, Mohammed T, Pretorius Holme M, Wang R, Kotokwe KP, et al. Cross-sectional estimates revealed high HIV incidence in Botswana rural communities in the era of successful ART scale-up in 2013–2015. *PLoS One*. 2018;13(10):e0204840. pmid:30356287

53.Psomas CK, Penaranda G, Retornaz F, Khiri H, Delord M, Halfon P, Philibert P. A cohort analysis of sexually transmitted infections among different groups of men who have sex with men in the early era of HIV pre-exposure prophylaxis in France. *J Virus Erad*. 2022 Feb 24;8(1):100065. doi: 10.1016/j.jve.2022.100065. PMID: 35251684; PMCID: PMC8891709.

54.Mushamiri I, Mnisi Z, Nkambule M, Justman J. A Longitudinal Analysis of the Impact of Health Behavior Campaigns on HIV-Risk Behaviors and HIV Incidence in Eswatini. *AIDS Behav*. 2021 Sep;25(9):2767-2778. doi: 10.1007/s10461-021-03316-7. Epub 2021 Aug 14. PMID: 34389891; PMCID: PMC8482811.

55.Schaefer, R., Gregson, S., Eaton, J. W., Mugurungi, O., Rhead, R., Takaruza, A., Maswera, R., & Nyamukapa, C. (2017). Age-disparate relationships and HIV incidence in adolescent girls and young women: evidence from Zimbabwe. *AIDS (London, England*), 31(10), 1461–1470. <https://doi.org/10.1097/QAD.0000000000001506>

56.Donnell D, Zewdie K, Ratna N, Miller V, Saunders JM, Gill ON, Delpech V, Mohammed H. Association between rectal gonorrhoea and HIV incidence in men who have sex with men: a meta-analysis. *Sex Transm Infect*. 2022 Nov;98(7):492-496. doi: 10.1136/sextrans-2021-055254. Epub 2021 Dec 15. PMID: 34911750; PMCID: PMC9613867.

57.Anwar S, El Kharrat E, Bakhoum A, El-Sadr WM, Harris TG. Association of sociodemographic factors with needle sharing and number of sex partners among people who inject drugs in Egypt. *Glob Public Health.* 2022 Aug;17(8):1689-1698. doi: 10.1080/17441692.2021.1950798. Epub 2021 Jul 20. PMID: 34283695.

58.Albert LM, Edwards J, Pence B, Speizer IS, Hillis S, Kahn K, Gómez-Olivé FX, Wagner RG, Twine R, Pettifor A. Associations of Father and Adult Male Presence with First Pregnancy and HIV Infection: Longitudinal Evidence from Adolescent Girls and Young Women in Rural South Africa (HPTN 068). *AIDS Behav.* 2021 Jul;25(7):2177-2194. doi: 10.1007/s10461-020-03147-y. Epub 2021 Jan 8. PMID: 33417105; PMCID: PMC8580262.

59.LeeVan E, Hu F, Mitchell AB, Kokogho A, Adebajo S, Garges EC, Qian H, Ake JA, Robb ML, Charurat ME, Baral SD, Nowak RG, Crowell TA; TRUST/RV368 Study Group. Associations of gender identity with sexual behaviours, social stigma and sexually transmitted infections among adults who have sex with men in Abuja and Lagos, Nigeria. *J Int AIDS Soc*. 2022 Jul;25(7):e25956. doi: 10.1002/jia2.25956. PMID: 35794838; PMCID: PMC9259961. Lagos, Nigeria doi: 10.1002/jia2.25956

60.Amelia de Sousa Mascena Veras M, Roza Saggese GS, Gomez Junior JL, Silveira P, Paiatto B, Ferreira D, Alves de Souza P, Calado R, Castejon MJ, Yamashiro R, Fernando de Macedo Brígido L, Turner C, Lin J, Wilson EC, McFarland W. Brief Report: Young Age and Sex Work Are Associated With HIV Seroconversion Among Transgender Women in São Paulo, Brazil. *J Acquir Immune Defic Syndr*. 2021 Sep 1;88(1):e1-e4. doi: 10.1097/QAI.0000000000002737. PMID: 34050104; PMCID: PMC8373663. 10.1097/QAI.0000000000002737

61.Javanbakht M, Miller AP, Moran A, Ragsdale A, Bolan R, Shoptaw S, Gorbach PM. Changes in Substance Use and Sexual Behaviors After a Sexually Transmitted Infection Diagnosis Among a Cohort of Men Who Have Sex With Men in Los Angeles, CA. *Sex Transm Dis.* 2023 Feb 1;50(2):112-120. doi: 10.1097/OLQ.0000000000001733. Epub 2022 Nov 6. PMID: 36342834; PMCID: PMC9839596.

62.Nyabuti MN, Petersen ML, Bukusi EA, Kamya MR, Mwangwa F, Kabami J, Sang N, Charlebois ED, Balzer LB, Schwab JD, Camlin CS, Black D, Clark TD, Chamie G, Havlir DV, Ayieko J. Characteristics of HIV seroconverters in the setting of universal test and treat: Results from the SEARCH trial in rural Uganda and Kenya. *PLoS One.* 2021 Feb 5;16(2):e0243167. doi: 10.1371/journal.pone.0243167. PMID: 33544717; PMCID: PMC7864429.

63.Chimbindi, N., Mthiyane, N., Birdthistle, I., Floyd, S., McGrath, N., Pillay, D., Seeley, J., Zuma, T., Dreyer, J., Gareta, D., Mutevedzi, T., Fenty, J., Herbst, K., Smit, T., Baisley, K., & Shahmanesh, M. (2018). Persistently high incidence of HIV and poor service uptake in adolescent girls and young women in rural KwaZulu-Natal, South Africa prior to DREAMS. *PloS one*, 13(10), e0203193. <https://doi.org/10.1371/journal.pone.0203193>

64.Ravindran J, Richardson BA, Kinuthia J, Unger JA, Drake AL, Osborn L, Matemo D, Patterson J, McClelland RS, John-Stewart G. Chlamydia, Gonorrhea, and Incident HIV Infection During Pregnancy Predict Preterm Birth Despite Treatment. *J Infect Dis*. 2021 Dec 15;224(12):2085-2093. doi: 10.1093/infdis/jiab277. PMID: 34023871; PMCID: PMC8672741.

65.Bhushan NL, Stoner MCD, Twine R, Kahn K, Lippman SA, Pettifor AE. Community Space, Community Groups, and Incident HIV Infection Among Adolescent Girls and Young Women in Rural South Africa: A Longitudinal Analysis of HIV Prevention Trials Network 068 Data. *J Acquir Immune Defic Syndr*. 2021 Jul 1;87(3):e207-e213. doi: 10.1097/QAI.0000000000002676. PMID: 33675621; PMCID: PMC8192416.

66.Nanthaprut P, Manojai N, Chanlearn P, Mattawanon N, Chiawkhun P, Homkham N, Traisathit P. Comparison of HIV-Positive Incidence Among Transgender Women and Men Who Have Sex with Men at Stand-Alone and Mobile Voluntary Counseling and Testing Facilities in Chiang Mai Province, Thailand. *AIDS Patient Care STDS*. 2021 Apr;35(4):116-125. doi: 10.1089/apc.2020.0258. PMID: 33835852.

67.van Santen DK, Lodi S, Dietze P, van den Boom W, Hayashi K, Dong H, Cui Z, Maher L, Hickman M, Boyd A, Prins M. Comprehensive needle and syringe program and opioid agonist therapy reduce HIV and hepatitis c virus acquisition among people who inject drugs in different settings: A pooled analysis of emulated trials. *Addiction.* 2023 Jun;118(6):1116-1126. doi: 10.1111/add.16147. Epub 2023 Feb 13. PMID: 36710474; PMCID: PMC10175130.

68.Samudyatha UC, Kosambiya JK, Patel HM. Cost-Effectiveness of Universal Repeat Human Immunodeficiency Virus Screening in Pregnancy: A Cross-Sectional Study from Western India. *Indian J Community Med.* 2021 Oct-Dec;46(4):668-672. doi: 10.4103/ijcm.IJCM_47_21. Epub 2021 Dec 8. PMID: 35068731; PMCID: PMC8729283.

69.Knox J, Hwang G, Carrico AW, Duncan DT, Watson RJ, Eaton LA. Daily Marijuana Use Predicts HIV Seroconversion Among Black Men Who Have Sex with Men and Transgender Women in Atlanta, GA. *AIDS Behav.* 2022 Aug;26(8):2503-2515. doi: 10.1007/s10461-022-03598-5. Epub 2022 Jan 30. PMID: 35094179; PMCID: PMC9720756.

70.Clipman SJ, Mehta SH, Mohapatra S, Srikrishnan AK, Zook KJC, Duggal P, Saravanan S, Nandagopal P, Kumar MS, Lucas GM, Latkin CA, Solomon SS. Deep learning and social network analysis elucidate drivers of HIV transmission in a high-incidence cohort of people who inject drugs. *Sci Adv*. 2022 Oct 21;8(42):eabf0158. doi: 10.1126/sciadv.abf0158. Epub 2022 Oct 19. PMID: 36260674; PMCID: PMC9581475.

71.Goin DE, Pearson RM, Craske MG, Stein A, Pettifor A, Lippman SA, Kahn K, Neilands TB, Hamilton EL, Selin A, MacPhail C, Wagner RG, Gomez-Olive FX, Twine R, Hughes JP, Agyei Y, Laeyendecker O, Tollman S, Ahern J. Depression and Incident HIV in Adolescent Girls and Young Women in HIV Prevention Trials Network 068: Targets for Prevention and Mediating Factors. *Am J Epidemiol*. 2020 May 5;189(5):422-432. doi: 10.1093/aje/kwz238. PMID: 31667490; PMCID: PMC7306677.

72.Willcox AC, Richardson BA, Shafi J, Kabare E, Kinuthia J, Jaoko W, Mandaliya K, Overbaugh J, McClelland RS. Derivation of an HIV Risk Score for African Women Who Engage in Sex Work. *AIDS Behav.* 2021 Oct;25(10):3292-3302. doi: 10.1007/s10461-021-03235-7. Epub 2021 Apr 16. PMID: 33861378; PMCID: PMC8763393.

73.Diabaté, S., Chamberland, A., Geraldo, N., Tremblay, C., & Alary, M. (2018). Gonorrhea, Chlamydia and HIV incidence among female sex workers in Cotonou, Benin: A longitudinal study. *PloS one*, *13*(5), e0197251. <https://doi.org/10.1371/journal.pone.0197251>

74.Wongjarupong N, Oli S, Sanou M, Djigma F, Kiba Koumare A, Yonli AT, Hassan MA, Mara K, Harmsen WS, Therneau T, Barro O, Vodounhessi G, Sawadogo S, Chamcheu JC, Simpore J, Roberts LR, Nagalo BM. Distribution and Incidence of Blood-Borne Infection among Blood Donors from Regional Transfusion Centers in Burkina Faso: A Comprehensive Study. *Am J Trop Med Hyg*. 2021 Feb 22;104(4):1577-1581. doi: 10.4269/ajtmh.20-0601. PMID: 33617474; PMCID: PMC8045619.

75.Negedu-Momoh OR, Balogun O, Dafa I, Etuk A, Oladele EA, Adedokun O, James E, Pandey SR, Khamofu H, Badru T, Robinson J, Mastro TD, Torpey K. Estimating HIV incidence in the Akwa Ibom AIDS indicator survey (AKAIS), Nigeria using the limiting antigen avidity recency assay. *J Int AIDS Soc.* 2021 Feb;24(2):e25669. doi: 10.1002/jia2.25669. PMID: 33619853; PMCID: PMC7900440.

76.Birdthistle I, Kwaro D, Shahmanesh M, Baisley K, Khagayi S, Chimbindi N, Kamire V, Mthiyane N, Gourlay A, Dreyer J, Phillips-Howard P, Glynn J, Floyd S. Evaluating the impact of DREAMS on HIV incidence among adolescent girls and young women: A population-based cohort study in Kenya and South Africa. *PLoS Med.* 2021 Oct 25;18(10):e1003837. doi: 10.1371/journal.pmed.1003837. PMID: 34695112; PMCID: PMC8880902.

77.Teixeira SL, Jalil CM, Jalil EM, Nazer SC, Silva SDCC, Veloso VG, Luz PM, Grinsztejn B. Evidence of an untamed HIV epidemic among MSM and TGW in Rio de Janeiro, Brazil: a 2018 to 2020 cross-sectional study using recent infection testing. *J Int AIDS Soc*. 2021 Jun;24(6):e25743. doi: 10.1002/jia2.25743. PMID: 34132470; PMCID: PMC8207443.

78.Konda KA, Torres TS, Mariño G, Ramos A, Moreira RI, Leite IC, Cunha M, Jalil EM, Hoagland B, Guanira JV, Benedetti M, Pimenta C, Vermandere H, Bautista-Arredondo S, Vega-Ramirez H, Veloso VG, Caceres CF, Grinsztejn B; ImPrEP Study Group. Factors associated with long-term HIV pre-exposure prophylaxis engagement and adherence among transgender women in Brazil, Mexico and Peru: results from the ImPrEP study. *J Int AIDS Soc.* 2022 Oct;25 Suppl 5(Suppl 5):e25974. doi: 10.1002/jia2.25974. PMID: 36225148; PMCID: PMC9557020.

79.Hui S, Chen F, Li Y, Cui Y, Zhang J, Zhang L, Yang Y, Liu Y, Zhao Y, Lv F. Factors Associated With Newly HIV Infection and Transmitted Drug Resistance Among Men Who Have Sex With Men in Harbin, P.R. China. *Front Public Health*. 2022 Jun 2;10:860171. doi: 10.3389/fpubh.2022.860171. PMID: 35719611; PMCID: PMC9201057.

80.Mutisya EM, Muturi-Kioi V, Abaasa A, Nyasani D, Kabuti RW, Lunani L, Kotikot T, Mundia M, Mutua G, Ombati G, Nduta H, Price MA, Kimani J, Anzala AO. Feasibility of conducting HIV prevention trials among key populations in Nairobi, Kenya. *BMC Public Health*. 2022 Dec 20;22(1):2385. doi: 10.1186/s12889-022-14875-2. PMID: 36536335; PMCID: PMC9762985.

81.Sturt AS, Webb EL, Phiri CR, Mudenda M, Mapani J, Kosloff B, Cheeba M, Shanaube K, Bwalya J, Kjetland EF, Francis SC, Corstjens PLAM, van Dam GJ, van Lieshout L, Hansingo I, Ayles H, Hayes RJ, Bustinduy AL. Female Genital Schistosomiasis and HIV-1 Incidence in Zambian Women: A Retrospective Cohort Study. *Open Forum Infect Dis.* 2021 Jun 30;8(7):ofab349. doi: 10.1093/ofid/ofab349. PMID: 34337098; PMCID: PMC8320261.

82.Samji H, Hu J, Otterstatter M, Hull M, Grennan T, Moore D, Gilbert M, Higgins R, Wong J. Gay, bisexual, and other men who have sex with men accessing STI clinics: Optimizing HIV PrEP implementation. *PLoS One*. 2022 Jan 27;17(1):e0261705. doi: 10.1371/journal.pone.0261705. PMID: 35085280; PMCID: PMC8794162.

83.Tunnage J, Yates A, Nwoga C, Sing'oei V, Owuoth J, Polyak CS, Crowell TA; RV393 Study Group. Hepatitis and tuberculosis testing are much less common than HIV testing among adults in Kisumu, Kenya: results from a cross-sectional assessment. *BMC Public Health*. 2021 Jun 15;21(1):1143. doi: 10.1186/s12889-021-11164-2. PMID: 34130663; PMCID: PMC8204299.

84.Skaathun, B.; Shrader, C. H.; Borquez, A.; Chaillon, A.; Vasylyeva, T.; Artamanova, I.; Harvey-Vera, A.; Rangel, G.; Strathdee, HIGH HIV INCIDENCE among PWID on the US/MEXICO BORDER during the COVID-19 PANDEMIC *S..Topics in Antiviral Medicine* ; 30(1 SUPPL):315-316, 2022.Article in English | EMBASE | ID: covidwho-1880880

85.Palanee-Phillips T, Rees HV, Heller KB, Ahmed K, Batting J, Beesham I, Heffron R, Justman J, Makkan H, Mastro TD, Morrison SA, Mugo N, Nair G, Kiarie J, Philip NM, Pleaner M, Reddy K, Selepe P, Steyn PS, Scoville CW, Smit J, Thomas KK, Donnell D, Baeten JM; ECHO Trial Consortium. High HIV incidence among young women in South Africa: Data from a large prospective study. *PLoS One*. 2022 Jun 3;17(6):e0269317. doi: 10.1371/journal.pone.0269317. PMID: 35657948; PMCID: PMC9165791.

86.Ssempijja V, Nakigozi G, Ssekubugu R, Kagaayi J, Kigozi G, Nalugoda F, Nantume B, Batte J, Kigozi G, Yeh PT, Nakawooya H, Serwadda D, Quinn TC, Gray RH, Wawer MJ, Grabowski KM, Chang LW, Van't Hoog A, Cobelens F, Reynolds SJ. High Rates of Pre-exposure Prophylaxis Eligibility and Associated HIV Incidence in a Population With a Generalized HIV Epidemic in Rakai, Uganda. *J Acquir Immune Defic Syndr.* 2022 Jul 1;90(3):291-299. doi: 10.1097/QAI.0000000000002946. Epub 2022 Mar 8. PMID: 35259129; PMCID: PMC9177156.

87.Ditangco R, Mationg ML. HIV incidence among men who have sex with men (MSM) in Metro Manila, the Philippines: A prospective cohort study 2014-2018. *Medicine (Baltimore).* 2022 Sep 2;101(35):e30057. doi: 10.1097/MD.0000000000030057. PMID: 36107537; PMCID: PMC9439796.

88.Lewis L, Kharsany ABM, Humphries H, Maughan-Brown B, Beckett S, Govender K, Cawood C, Khanyile D, George G. HIV incidence and associated risk factors in adolescent girls and young women in South Africa: A population-based cohort study. *PLoS One*. 2022 Dec 21;17(12):e0279289. doi: 10.1371/journal.pone.0279289. PMID: 36542645; PMCID: PMC9770356.

89.Lambert G, Cox J, Fourmigue A, Dvorakova M, Apelian H, Moodie EEM, Grace D, Skakoon-Sparling S, Moore DM, Lachowsky N, Jollimore J, Lal A, Parlette A, Hart TA; Engage Study Team. HIV incidence and related risks among gay, bisexual, and other men who have sex with men in Montreal, Toronto, and Vancouver: Informing blood donor selection criteria in Canada. *Transfusion*. 2022 Dec;62(12):2555-2567. doi: 10.1111/trf.17127. Epub 2022 Oct 5. PMID: 36197064; PMCID: PMC10092181.

90.Shan D, Ning Z, Yu M, Zheng H, Yang J, Gong H, Li J, Liu H, Liu L, Wang V, Ran X, Han M, Zhang D. HIV incidence and risk factors among transgender women and cisgender men who have sex with men in two cities of China: a prospective cohort study. *Infect Dis Poverty*. 2022 Mar 7;11(1):26. doi: 10.1186/s40249-022-00947-3. PMID: 35256001; PMCID: PMC8900389.

91.Sandfort TGM, Mbilizi Y, Sanders EJ, Guo X, Cummings V, Hamilton EL, Akelo V, Panchia R, Dominguez K, Stirratt MJ, Chege W, Lucas J, Gaydos CA, Chen YQ, Eshleman SH. HIV incidence in a multinational cohort of men and transgender women who have sex with men in sub-Saharan Africa: Findings from HPTN 075. *PLoS One*. 2021 Feb 25;16(2):e0247195. doi: 10.1371/journal.pone.0247195.

92.Ussery F, Bachanas P, Alwano MG, Lebelonyane R, Block L, Wirth K, Ussery G, Sento B, Gaolathe T, Kadima E, Abrams W, Segolodi T, Hader S, Lockman S, Moore J. HIV Incidence in Botswana Rural Communities With High Antiretroviral Treatment Coverage: Results From the Botswana Combination Prevention Project, 2013-2017. *J Acquir Immune Defic Syndr*. 2022 Sep 1;91(1):9-16. doi: 10.1097/QAI.0000000000003017. PMID: 35537094; PMCID: PMC9388617.

93.Nkambule R, Philip NM, Reid G, Mnisi Z, Nuwagaba-Biribonwoha H, Ao TT, Ginindza C, Duong YT, Patel H, Saito S, Solmo C, Brown K, Moore CS, Voetsch AC, Bicego G, Bock N, Mhlanga F, Dlamini T, Mabuza K, Zwane A, Sahabo R, Dobbs T, Parekh BS, El-Sadr W, Ryan C, Justman J. HIV incidence, viremia, and the national response in Eswatini: Two sequential population-based surveys. *PLoS One*. 2021 Dec 2;16(12):e0260892. doi: 10.1371/journal.pone.0260892. PMID: 34855890; PMCID: PMC8639055.

94.van Griensven F, Phanuphak N, Manopaiboon C, Dunne EF, Colby DJ, Chaiphosri P, Ramautarsing R, Mock PA, Guadamuz TE, Rangsin R, Benjamaneepairoj K, Na Nakorn P, Vannakit R, de Lind van Wijngaarden JW, Avery M, Mills S. HIV prevalence and incidence among men who have sex with men and transgender women in Bangkok, 2014-2018: Outcomes of a consensus development initiative. *PLoS One*. 2022 Jan 21;17(1):e0262694. doi: 10.1371/journal.pone.0262694. PMID: 35061803; PMCID: PMC8782340.

95.Li M, Wu H, Yan H, Zunong J, Hui H, Li H, Yang Z, Vermund SH, Hu Y. HIV-Positive Men Are More Likely to Be Hyper Linked Within College Student Social Network - Northeast China, 2017-2018. *China CDC Wkly.* 2022 Oct 28;4(43):951-955. doi: 10.46234/ccdcw2022.195. PMID: 36483793; PMCID: PMC9713575.

96.Steele WR, Dodd RY, Notari EP, Haynes J, Anderson SA, Williams AE, Reik R, Kessler D, Custer B, Stramer SL; Transfusion-Transmissible Infections Monitoring System (TTIMS). HIV, HCV, and HBV incidence and residual risk in US blood donors before and after implementation of the 12-month deferral policy for men who have sex with men. *Transfusion*. 2021 Mar;61(3):839-850. doi: 10.1111/trf.16250. Epub 2021 Jan 18. PMID: 33460470.

97.Luz E, Marques M, Netto EM, Campos LM, Amaral S, Santana I, Marques EL, Brites C. HIV, HTLV, and Hepatitis B and C Infection in Blood Donors in Bahia, Brazil from 2008 to 2017. *Viruses*. 2022 Oct 22;14(11):2323. doi: 10.3390/v14112323. PMID: 36366422; PMCID: PMC9692744.

98.Björkman Nyqvist M, Corno L, de Walque D, Svensson J. HIV, risk, and time preferences: Evidence from a general population sample in Lesotho. *Health Econ.* 2022 May;31(5):904-911. doi: 10.1002/hec.4476. Epub 2022 Feb 11. PMID: 35150024.

99.Penumetsa M, Neary J, Farid S, Kithao P, Richardson BA, Matemo D, John-Stewart G, Kinuthia J, Drake AL. Implementation of HIV Retesting During Pregnancy and Postpartum in Kenya: A Cross-Sectional Study. *Glob Health Sci Pract*. 2022 Feb 28;10(1):e2100451. doi: 10.9745/GHSP-D-21-00451. PMID: 35294386; PMCID: PMC8885347.

100.Nouaman MN, Becquet V, Plazy M, Coffie PA, Zébago C, Montoyo A, Anoma C, Eholié S, Dabis F, Larmarange J; ANRS 12361 PrEP-CI Study group. Incidence of HIV infection and associated factors among female sex workers in Côte d'Ivoire, results of the ANRS 12361 PrEP-CI study using recent infection assays. *PLoS One*. 2022 Nov 17;17(11):e0271988. doi: 10.1371/journal.pone.0271988. PMID: 36395099; PMCID: PMC9671321.

101.Mussa A, Mayondi GK, Diseko M, Mabuta J, Mmalane M, Makhema J, Lockman S, Morroni C, Shapiro R, Zash R. Incident HIV acquisition among pregnant women in Botswana: findings from the Tsepamo birth outcomes surveillance study. *J Int AIDS Soc.* 2023 Jan;26(1):e26008. doi: 10.1002/jia2.26008. PMID: 36691796; PMCID: PMC9871722.

102.Hoffman S, Zhang A, Nguyen N, Tsong R, Chen IS, Wei Y, Lutalo T, Nalugoda F, Kennedy CE, Grabowski MK, Santelli JS. Incident HIV Infection Among Young Men Associated With Female Sexual Partner Types Identified Through Latent Class Analysis, Rakai, Uganda. *J Acquir Immune Defic Syndr*. 2022 Jun 1;90(2):124-131. doi: 10.1097/QAI.0000000000002928. PMID: 35125472; PMCID: PMC9203866.

103.Abrahams N, Mhlongo S, Dunkle K, Chirwa E, Lombard C, Seedat S, Kengne AP, Myers B, Peer N, Garcia-Moreno C, Jewkes R. Increase in HIV incidence in women exposed to rape*. AIDS*. 2021 Mar 15;35(4):633-642. doi: 10.1097/QAD.0000000000002779. PMID: 33264114; PMCID: PMC7924974.

104.Jones HS, Hensen B, Musemburi S, Chinyanganya L, Takaruza A, Chabata ST, Matambanadzo P, Rice B, Cowan FM, Hargreaves JR. Interpreting declines in HIV test positivity: an analysis of routine data from Zimbabwe's national sex work programme, 2009-2019. *J Int AIDS Soc*. 2022 Jul;25(7):e25943. doi: 10.1002/jia2.25943. PMID: 35773959; PMCID: PMC9247303.

105.Jain JP, Abramovitz D, Strathdee SA, Gonzalez-Zuniga P, Rangel G, West BS, Pitpitan EV. Sex Work as a Mediator Between Female Gender and Incident HIV Infection Among People Who Inject Drugs in Tijuana, Mexico. *AIDS Behav*. 2020 Sep;24(9):2720-2731. doi: 10.1007/s10461-020-02828-y. PMID: 32185596; PMCID: PMC7453791.

106.Kilburn, K., Ranganathan, M., Stoner, M. C. D., Hughes, J. P., MacPhail, C., Agyei, Y., Gómez-Olivé, F. X., Kahn, K., & Pettifor, A. (2018). Transactional sex and incident HIV infection in a cohort of young women from rural South Africa. *AIDS (London, England)*, *32*(12), 1669–1677. <https://doi.org/10.1097/QAD.0000000000001866>

107.Kritsanavarin U, Bloss E, Manopaiboon C, Khawcharoenporn T, Harnlakon P, Vasanti-Uppapokakorn M, Kitwattanachai P, Naprasert S, Phiphatthananon T, Visavakum P, Jetsawang B, Mock PA. HIV incidence among men who have sex with men and transgender women in four provinces in Thailand. *Int J STD AIDS*. 2020 Oct;31(12):1154-1160. doi: 10.1177/0956462420921068. Epub 2020 Sep 9. PMID: 32903141; PMCID: PMC9792256.

108.Akullian A, Vandormael A, Miller JC, Bershteyn A, Wenger E, Cuadros D, Gareta D, Bärnighausen T, Herbst K, Tanser F. Large age shifts in HIV-1 incidence patterns in KwaZulu-Natal, South Africa*. Proc Natl Acad Sci U S A*. 2021 Jul 13;118(28):e2013164118. doi: 10.1073/pnas.2013164118. PMID: 34244424; PMCID: PMC8285891.

109.Machekano, R., Tiam, A., Kassaye, S., Tukei, V., Gill, M., Mohai, F., Nchepe, M., Mokone, M., Barasa, J., Mohale, S., Letsie, M., & Guay, L. (2018). HIV incidence among pregnant and postpartum women in a high prevalence setting. *PloS one*, *13*(12), e0209782. <https://doi.org/10.1371/journal.pone.0209782>

110.Evans KN, Vettese T, Wortley PM, Gandhi AP, Bradley H. Missed opportunities for prevention: prevalence and incidence of human immunodeficiency virus and hepatitis C virus diagnoses among a cohort of individuals discharged from an urban hospital with injection drug-related diagnoses, 2012-2019. *Ann Epidemiol*. 2023 Apr;80:69-75.e2. doi: 10.1016/j.annepidem.2023.02.005. Epub 2023 Feb 13. PMID: 36791871.

111.Nikolopoulos GK, Chanos S, Tsioptsias E, Hodges-Mameletzis I, Paraskeva D, Dedes N. HIV incidence among men who have sex with men at a community-based facility in Greece. *Cent Eur J Public Health.* 2019 Mar;27(1):54-57. doi: 10.21101/cejph.a4856. PMID: 30927398.

112.Lee YC, Liu WC, Hsieh YL, Wu CH, Wu PY, Luo YZ, Yang JY, Chen YH, Fang CT, Hung CC, Chang SC. Non-opioid recreational drug use and a prolonged HIV outbreak among men who have sex with men in Taiwan: An incident case-control study, 2006-2015. *J Formos Med Assoc*. 2022 Jan;121(1 Pt 2):237-246. doi: 10.1016/j.jfma.2021.03.015. Epub 2021 Apr 4. PMID: 33824010.

113.Nowak, R. G., Mitchell, A., Crowell, T. A., Liu, H., Ketende, S., Ramadhani, H. O., Ndembi, N., Adebajo, S., Ake, J., Michael, N. L., Blattner, W. A., Baral, S. D., & Charurat, M. E. (2019). Individual and Sexual Network Predictors of HIV Incidence Among Men Who Have Sex With Men in Nigeria. *Journal of acquired immune deficiency syndromes (1999)*, *80*(4), 444–453. <https://doi.org/10.1097/QAI.0000000000001934>

114.Roussos S, Paraskevis D, Psichogiou M, Kostaki EG, Flountzi E, Angelopoulos T, Chaikalis S, Papadopoulou M, Pavlopoulou ID, Malliori M, Hatzitheodorou E, Pylli M, Tsiara C, Paraskeva D, Beloukas A, Kalamitsis G, Hatzakis A, Sypsa V. Ongoing HIV transmission following a large outbreak among people who inject drugs in Athens, Greece (2014-20). *Addiction*. 2022 Jun;117(6):1670-1682. doi: 10.1111/add.15812. Epub 2022 Feb 21. PMID: 35072299.

115.Piyaraj P, van Griensven F, Holtz TH, Mock PA, Varangrat A, Wimonsate W, Thienkrua W, Tongtoyai J, McNamara A, Chonwattana W, Nelson KE. The finding of casual sex partners on the internet, methamphetamine use for sexual pleasure, and incidence of HIV infection among men who have sex with men in Bangkok, Thailand: an observational cohort study. *Lancet HIV*. 2018 Jul;5(7):e379-e389. doi: 10.1016/S2352-3018(18)30065-1. Epub 2018 Jun 1.

116.Celum CL, Bukusi EA, Bekker LG, Delany-Moretlwe S, Kidoguchi L, Omollo V, Rousseau E, Travill D, Morton JF, Mogaka F, O'Malley G, Barnabee G, van der Straten A, Donnell D, Parikh UM, Kudrick L, Anderson PL, Haberer JE, Wu L, Heffron R, Johnson R, Morrison S, Baeten JM; POWER Study Team. PrEP use and HIV seroconversion rates in adolescent girls and young women from Kenya and South Africa: the POWER demonstration project. *J Int AIDS Soc*. 2022 Jul;25(7):e25962. doi: 10.1002/jia2.25962. PMID: 35822945; PMCID: PMC9278271.

117.Hoque M, Hoque ME, van Hal G, Buckus S. Prevalence, incidence and seroconversion of HIV and Syphilis infections among pregnant women of South Africa. *S Afr J Infect Dis*. 2021 Nov 24;36(1):296. doi: 10.4102/sajid.v36i1.296. PMID: 34917677; PMCID: PMC8661397.

118.Woldesenbet, S., Kufa-Chakezha, T., Lombard, C., Manda, S., Cheyip, M., Ayalew, K., Chirombo, B., Barron, P., Diallo, K., Parekh, B., & Puren, A. (2021). Recent HIV infection among pregnant women in the 2017 antenatal sentinel cross-sectional survey, South Africa: Assay-based incidence measurement. *PloS one*, *16*(4), e0249953. <https://doi.org/10.1371/journal.pone.0249953>

119.Mohloanyane T, Olivier D, Labhardt ND, Amstutz A. Recent HIV infections among newly diagnosed individuals living with HIV in rural Lesotho: Secondary data from the VIBRA cluster-randomized trial. *PLoS One*. 2022 Nov 21;17(11):e0277812. doi: 10.1371/journal.pone.0277812. PMID: 36409754; PMCID: PMC9678280.

120.Rwibasira, G. N., Malamba, S. S., Musengimana, G., Nkunda, R. C. M., Omolo, J., Remera, E., Masengesho, V., Mbonitegeka, V., Dzinamarira, T., Kayirangwa, E., & Mugwaneza, P. (2021). Recent infections among individuals with a new HIV diagnosis in Rwanda, 2018-2020. *PloS one*, *16*(11), e0259708. <https://doi.org/10.1371/journal.pone.0259708>

121.Gras J, Pillet M, Antoni G, Cua E, Charreau I, Raffi F, Chidiac C, Chas J, Tremblay C, Spire B, Delaugerre C, Meyer L, Molina JM; IPERGAY Study group. Risk factors for HIV infection among men who have sex with men in the ANRS IPERGAY PrEP trial. *Sex Transm Infect.* 2022 Aug;98(5):383-386. doi: 10.1136/sextrans-2021-055199. Epub 2021 Oct 18. PMID: 34663696.

122.Mayer ME, White E, Montano MA, Lama JR, Sanchez H, Cabello R, Sanchez J, Pasalar S, Duerr A. Sexual Behavior Among Men Who Have Sex With Men: The Need for More Targeted Outreach to Men Who Also Have Sex With Cisgender Women. *J Acquir Immune Defic Syndr*. 2021 Mar 1;86(3):265-270. doi: 10.1097/QAI.0000000000002568. PMID: 33148993; PMCID: PMC8605625.

123.Yu Z, Zhang TT, Wang X, Chang Q, Huang H, Zhang H, Song D, Yu M, Yang J, Liu Y, Li C, Cui Z, Ma J. Sexual behaviour changes and HIV infection among men who have sex with men: evidence from an open cohort in China. *BMJ Open*. 2022 Sep 28;12(9):e055046. doi: 10.1136/bmjopen-2021-055046. PMID: 36171031; PMCID: PMC9528664.

124.Rosa PB, Schroeder DF, Darsie C, Chielle M, Pontel MLK, Correa GB, Vilichane IJ, Heringer TA, Renner JDP, Possuelo LG. Spatial distribution and incidence of HIV/AIDS cases in Santa Cruz do Sul, state of Rio Grande do Sul, 2001 to 2020. *Epidemiol Serv Saude*. 2022 Dec 19;31(3):e2022323. doi: 10.1590/S2237-96222022000300020. PMID: 36542044; PMCID: PMC9887989.

125.Justman J, Reed JB, Bicego G, Donnell D, Li K, Bock N, Koler A, Philip NM, Mlambo CK, Parekh BS, Duong YT, Ellenberger DL, El-Sadr WM, Nkambule R. Swaziland HIV Incidence Measurement Survey (SHIMS): a prospective national cohort study. *Lancet HIV*. 2017 Feb;4(2):e83-e92. doi: 10.1016/S2352-3018(16)30190-4. Epub 2016 Nov 16. PMID: 27863998; PMCID: PMC5291824.

126.Patel EU, Solomon SS, Lucas GM, McFall AM, Srikrishnan AK, Kumar MS, Iqbal SH, Saravanan S, Paneerselvam N, Balakrishnan P, Laeyendecker O, Celentano DD, Mehta SH. Temporal change in population-level prevalence of detectable HIV viraemia and its association with HIV incidence in key populations in India: a serial cross-sectional study. *Lancet HIV*. 2021 Sep;8(9):e544-e553. doi: 10.1016/S2352-3018(21)00098-9. Epub 2021 Jul 28. PMID: 34331860; PMCID: PMC9164229.

127.Wand, H., Reddy, T., & Ramjee, G. (2021). Temporal trends in sexual behaviours and their impacts on HIV incidence among South African women: 2002-2016. *AIDS care*, *33*(8), 1002–1008. <https://doi.org/10.1080/09540121.2020.1789054>

128.Mthiyane N, Baisley K, Chimbindi N, Zuma T, Okesola N, Dreyer J, Herbst C, Smit T, Danaviah S, McGrath N, Harling G, Sherr L, Seeley J, Floyd S, Birdthistle I, Shahmanesh M. The association of exposure to DREAMS on sexually acquiring or transmitting HIV amongst adolescent girls and young women living in rural South Africa. *AIDS*. 2022 Jun 15;36(Suppl 1):S39-S49. doi: 10.1097/QAD.0000000000003156. PMID: 35766574.

129.Moses SJ, Wachekwa I, Van Ryn C, Grandits G, Pau A, Badio M, Kennedy SB, Sneller MC, Higgs ES, Lane HC, Fallah M, Migueles SA, Reilly C. The impact of the 2014 Ebola epidemic on HIV disease burden and outcomes in Liberia West Africa. *PLoS One*. 2021 Sep 10;16(9):e0257049. doi: 10.1371/journal.pone.0257049. PMID: 34506540; PMCID: PMC8432817.

130.Faini D, Msafiri F, Munseri P, Bakari M, Lyamuya E, Sandström E, Biberfeld G, Nilsson C, Hanson C, Aboud S. The Prevalence, Incidence, and Risk Factors for HIV Among Female Sex Workers-A Cohort Being Prepared for a Phase IIb HIV Vaccine Trial in Dar es Salaam, Tanzania. *J Acquir Immune Defic Syndr.* 2022 Dec 15;91(5):439-448. doi: 10.1097/QAI.0000000000003097. Epub 2022 Sep 20. PMID: 36126184; PMCID: PMC9646411.

131.Sudenga SL, Lotspeich SC, Nyitray AG, Sirak B, Shepherd BE, Messina J, Sereday KA, Silva RC, Abrahamsen M, Baggio ML, Quiterio M, Lazcano-Ponce E, Villa L, Giuliano AR. The Role of External Genital Lesions in Human Immunodeficiency Virus Seroconversion Among Men Participating in a Multinational Study. *Sex Transm Dis.* 2022 Jan 1;49(1):55-58. doi: 10.1097/OLQ.0000000000001516. PMID: 34282740; PMCID: PMC8722569.

132.Thienkrua W, van Griensven F, Mock PA, Dunne EF, Raengsakulrach B, Wimonsate W, Howteerakul N, Ungsedhapand C, Chiwarakorn A, Holtz TH. Young Men Who Have Sex with Men at High Risk for HIV, Bangkok MSM Cohort Study, Thailand 2006-2014. *AIDS Behav*. 2018 Jul;22(7):2137-2146. doi: 10.1007/s10461-017-1963-7. PMID: 29138981; PMCID: PMC6409204.

133.Wand, H., Morris, N. & Reddy, T. Understanding the public health implications of self-reported condom use in HIV clinical trials: lessons learned in KwaZulu Natal, South Africa (2002–2016). *J Public Health (Berl.)* (2021). <https://doi.org/10.1007/s10389-021-01639-2>

134.van Griensven F, Janamnuaysook R, Nampaisan O, Peelay J, Samitpol K, Mills S, Pankam T, Ramautarsing R, Teeratakulpisarn N, Phanuphak P, Phanuphak N. Uptake of Primary Care Services and HIV and Syphilis Infection among Transgender Women attending the Tangerine Community Health Clinic, Bangkok, Thailand, 2016 - 2019. *J Int AIDS Soc*. 2021 Jun;24(6):e25683. doi: 10.1002/jia2.25683. PMID: 34152695; PMCID: PMC8216133.

135.Laher F, Otwombe K, Mokwena O, Bekker LG, Allen M. Use of Varied Screening Risk Criteria and HIV Incidence in Phase 1 and 2 HIV Vaccine Trials in South Africa. *AIDS Behav.* 2023 Apr;27(4):1314-1320. doi: 10.1007/s10461-022-03867-3. Epub 2022 Oct 26. PMID: 36287343; PMCID: PMC10038814.

136.Des Jarlais DC, Arasteh K, Huong DT, Oanh KTH, Feelemyer JP, Khue PM, Giang HT, Thanh NTT, Vinh VH, Le SM, Vallo R, Quillet C, Rapoud D, Michel L, Laureillard D, Moles JP, Nagot N; DRIVE Study Team. Using large-scale respondent driven sampling to monitor the end of an HIV epidemic among persons who inject drugs in Hai Phong, Viet Nam. *PLoS One*. 2021 Nov 18;16(11):e0259983. doi: 10.1371/journal.pone.0259983. PMID: 34793523; PMCID: PMC8601441.

137.Ortblad KF, Mawandia S, Bakae O, Tau L, Grande M, Mogomotsi GP, Mmatli E, Ngombo M, Seckel L, Heffron R, Pintye J, Ledikwe J. Using routine programmatic data to measure HIV incidence among pregnant women in Botswana. *Popul Health Metr.* 2022 Mar 4;20(1):10. doi: 10.1186/s12963-022-00287-2. PMID: 35246143; PMCID: PMC8896233.

138.You, X., Gilmour, S., Cao, W., Lau, J. T., Hao, C., Gu, J., Le, P. M., Peng, L., Wei, D., Deng, Y., Wang, X., Zou, H., Li, J., Hao, Y., & Li, J. (2021). HIV incidence and sexual behavioral correlates among 4578 men who have sex with men (MSM) in Chengdu, China: a retrospective cohort study. *BMC public health*, *21*(1), 802. <https://doi.org/10.1186/s12889-021-10835-4>

**Appendix 4: PRISMA Flow Diagram**

***A PRISMA Flow Diagram detailing the Systematic Review methodology and reasons for study exclusion.***

Studies identified through reference screening and full-text screened

(n = 41)

**Identification**

Studies identified through database searching

(n = 45,264)

Studies removed after title/abstract screening

(n = 13,354)

**Screening**

Studies removed after full-text screening, with reasons

(n = 14)

9 Wrong Study Design

3 Modelling Study

2 Variables Missing

Studies title/abstract screened (after duplicates removed)

(n = 13,733)

Studies removed after full-text screening, with reasons

(n = 308)

88 Wrong Outcomes

68 Wrong Study Design

65 Modelling Study

38 Variables Missing

30 Wrong Study Population

19 Full Text Not Available

Studies screened (full-text)

(n = 379)

**Eligibility**

**Included**

Studies included in data analysis

(n = 98)

**Appendix 5: Maths for Efficacy Estimates**

The 90% estimated efficacy for CAB-LA is calculated based on the formula:

*(1-IC) / (IP)*

*Where IC is the combined infection rate in the CAB-LA arms of the HPTN 083 and 084 trials and IP is the combined infection rate in the placebo arms of the iPrEx and IPERGAY trials*

This estimate is in line with studies estimating CAB-LA efficacy. A similar formula was used to estimate the 60% efficacy of continuous TDF/FTC:

*(1-IT) / (IPL)*

*Where IT is the combined infection rate in the TDF/FTC arms of the iPrEx, IPERGAY, TDF2 and PartnersPREP trials and IPL is the combined infection rate in the placebo arms of the iPrEx, IPERGAY, TDF2 and PartnersPREP trials.*

Inclusion of the iPrEx, IPERGAY, TDF2 and PartnersPREP trial results for calculation for efficacy was deemed reliable as per quality appraisal of studies conducted on TDF/FTC since 2010, corroborated by other systematic reviews^a, b.^

The 30% efficacy for event-driven TDF/FTC is a conservative estimate, as efficacy has not been measured outside of a trial context in risk populations. Therefore, the conservative estimate accounts for a potential reduction in efficacy during implementation in the community.

**References:**

(a) Pilkington, V., Hill, A., Hughes, S., Nwokolo, N., & Pozniak, A. (2018). How safe is TDF/FTC as PrEP? A systematic review and meta-analysis of the risk of adverse events in 13 randomised trials of PrEP. *Journal of virus eradication*, *4*(4), 215–224.

(b) Huang, X., Hou, J., Song, A., Liu, X., Yang, X., Xu, J., Zhang, J., Hu, Q., Chen, H., Chen, Y., Meyers, K., & Wu, H. (2018). Efficacy and Safety of Oral TDF-Based Pre-exposure Prophylaxis for Men Who Have Sex With Men: A Systematic Review and Meta-Analysis. *Frontiers in pharmacology*, *9*, 799. https://doi.org/10.3389/fphar.2018.00799

**Appendix 6: Global Incidence**

***Table showing the number of studies*, total sample size, and average incidence of HIV infection in key populations, blood donors, and the general population.***

****Some studies provided data on more than one population.***

*
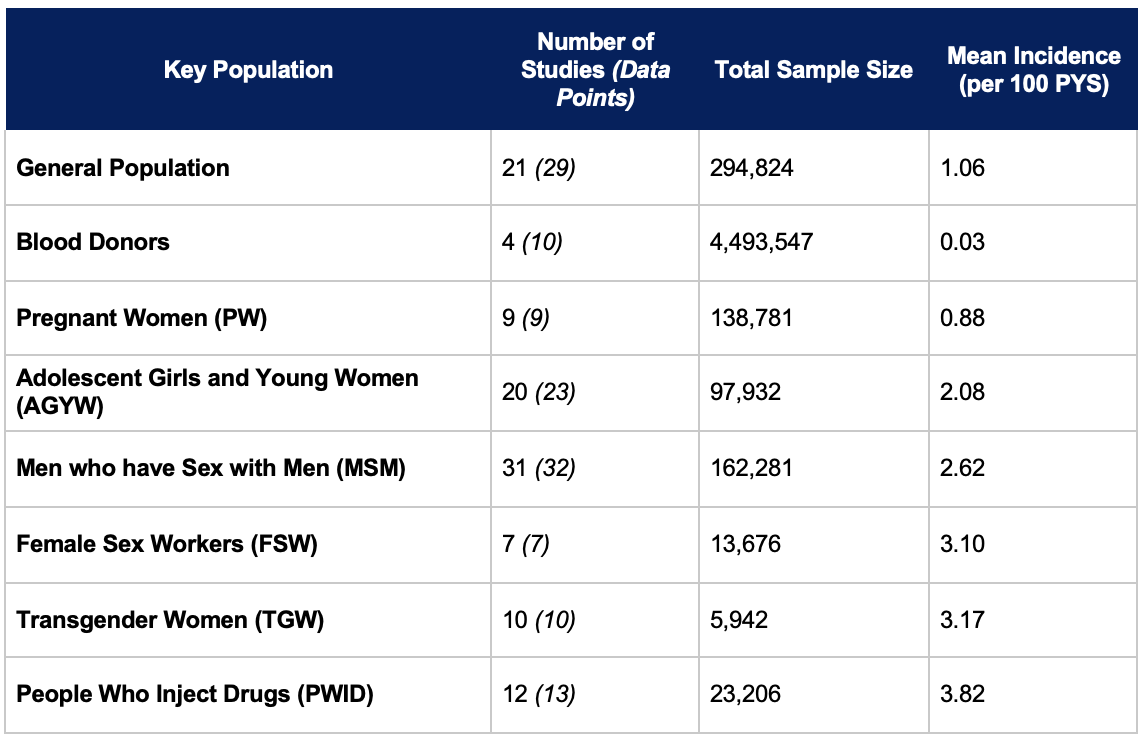
*

**Appendix 7: Incidence in Southern and East** **Africa**

***Table showing the number of studies*, total sample size, and average incidence of HIV infection in key populations, and the general population in Southern and East Africa.***

****Some studies provided data on more than one population.***

*
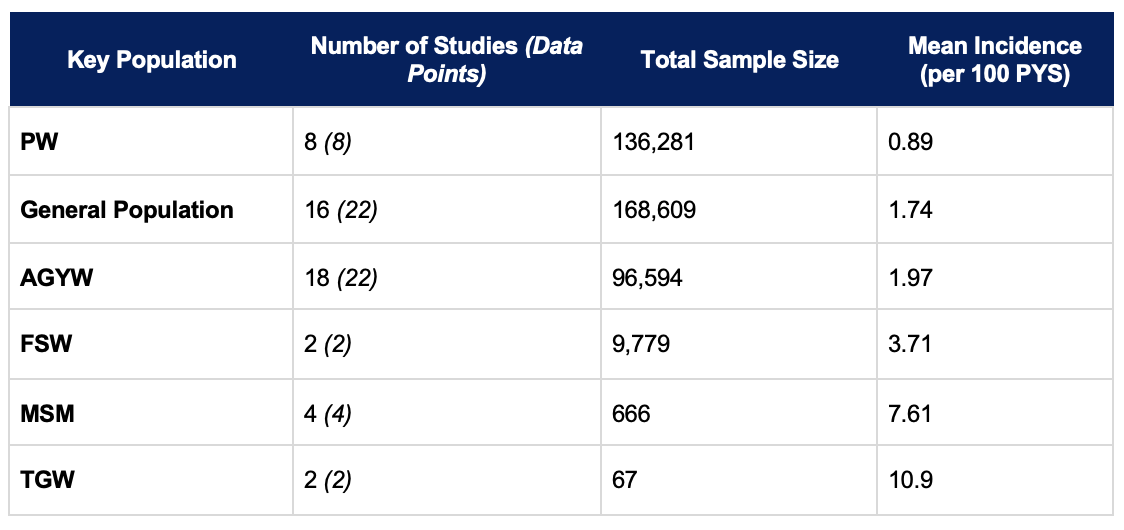
*
